# Identification and characterization of alternative exon usage linked glioblastoma multiforme survival

**DOI:** 10.1186/1755-8794-5-59

**Published:** 2012-12-04

**Authors:** Ahmed Sadeque, Nicola VL Serão, Bruce R Southey, Kristin R Delfino, Sandra L Rodriguez-Zas

**Affiliations:** 1 Department of Animal Sciences, University of Illinois Urbana-Champaign, Urbana, IL, USA; 2 Department of Statistics, University of Illinois Urbana-Champaign, Urbana, IL, USA; 3 Institute of Genomic Biology, University of Illinois Urbana-Champaign, Urbana, IL, USA

**Keywords:** Alternative Splicing (AS), Alternative Exon Usage (AEU), Glioblastoma Multiforme (GBM), Hierarchical model

## Abstract

**Background:**

Alternative exon usage (AEU) is an important component of gene regulation. Exon expression platforms allow the detection of associations between AEU and phenotypes such as cancer. Numerous studies have identified associations between gene expression and the brain cancer glioblastoma multiforme (GBM). The few consistent gene expression biomarkers of GBM that have been reported may be due to the limited consideration of AEU and the analytical approaches used. The objectives of this study were to develop a model that accounts for the variations in expression present between the exons within a gene and to identify AEU biomarkers of GBM survival.

**Methods:**

The expression of exons corresponding to 25,403 genes was related to the survival of 250 individuals diagnosed with GBM in a training data set. Genes exhibiting AEU in the training data set were confirmed in an independent validation data set of 78 patients. A hierarchical mixed model that allows the consideration of covariation between exons within a gene and of the effect of the epidemiological characteristics of the patients was developed to identify associations between exon expression and patient survival. This general model describes all three possible scenarios: multi-exon genes with and without AEU, and single-exon genes.

**Results:**

AEU associated with GBM survival was identified on 2477 genes (P-value < 5.0E-04 or FDR-adjusted P-value < 0.05). G-protein coupled receptor 98 (*Gpr98*) and epidermal growth factor (*Egf*) were among the genes exhibiting AEU with 30 and 9 exons associated with GBM survival, respectively. Pathways enriched among the AEU genes included focal adhesion, ECM-receptor interaction, ABC transporters and pathways in cancer. In addition, 24 multi-exon genes without AEU and 8 single-exon genes were associated with GBM survival (FDR-adjusted P-value < 0.05).

**Conclusions:**

The inferred patterns of AEU were consistent with *in silico* AS models. The hierarchical model used offered a flexible and simple way to interpret and identify associations between survival that accommodates multi-exon genes with or without AEU and single exon genes. Our results indicate that differential expression of AEU could be used as biomarker for GBM and potentially other cancers.

## Background

Alternative splicing (AS) is characterized by the formation of different mRNA isoforms as a result of including or excluding different exonic or intronic segments. This process is responsible for generating protein diversity from a finite number of genes [1-3]. Alternative splicing can be divided into three broad categories; intron retention, cryptic splice-site usage, and alternative exon usage (AEU) or exon skipping. Alternative exon usage includes cassette exons, which are discrete exons that can be independently included or excluded, and mutually exclusive splicing, which involves the selection of one from a group of exon variants [2]. Approximately 75% of multi-exon genes exhibit AS in humans [4]. The human genome includes approximately 28,526 annotated genes that express approximately 120,145 transcripts, of which 80,932 are protein coding and 39,213 are non-coding transcripts [5]. The identification of "exon-level" expression profiles and characterization of AS events has become possible with the availability of exon platforms (e.g. GeneChip Exon Array). The brain exhibits particularly high rates of AS [6] and the highest number of AEU events [7]. Regulation of gene expression due to splicing has been associated with cancer. Many AEU events have been associated with disordered cell differentiation and signaling that contribute to stem cell like proliferation of cancer cells [8].

Glioblastoma multiforme (GBM) is an aggressive type of brain cancer and the role of genes and AEU on GBM survival is still not completely understood [9-11]. Most work on AS and GBM studied individual genes or compared AS between GBM and control (e.g. blood) samples. The relationship between AS and the survival of individuals diagnosed with GBM has not been studied. Understanding of the factors influencing survival is particularly important in GBM cases because the median survival after diagnosis is approximately one year [12,13]. Furthermore, several epidemiological factors influence GBM survival including gender, race and treatment [14]. Thus, a more accurate understanding of the relationship between AS and GBM survival must consider epidemiological factors and inter-individual variability.

Several approaches to identify AS events have been proposed. However, most approaches have limitations that can bias the identification and characterization of AEU. For example, Su et al. developed an individual exon approach that does not model the covariation between exons within a gene [15]. Purdom et al. used the residuals from probe level analysis to identify AEU on a per-sample level [16]. The sample-level analysis challenges the detection of AEU events or the identification of common patterns across patients receiving the same treatment or from the same epidemiological strata. Laderas et al. and Zheng et al. proposed group comparison using linear models to overcome the limitations of the previous approach [2,17]. However, group comparison is not suited to identify AEU associated with other conditions such as survival or time-to-event. In addition, the previous implementation does not account for correlations between exons measured on the same sample. Cline et al. formulated an ANalysis Of Splice VAriation approach that cannot be used in genes that produced more than one splice form [18].

The main goal of this study is to demonstrate an exon-based, gene-centric model to detect AEU events associated with GBM survival. We developed an analytical method that addresses the limitations of previous approaches by modeling the exon-level expression profiles within genes from all samples across all treatments or conditions studied. Our approach accommodates the dependencies between exons within a gene and patient and allows testing the hypothesis of differential exon expression or usage between treatment groups. A unique advantage of our flexible approach is that one model encompasses all scenarios: i) multi-exon genes that have AEU, ii) multi-exon genes that do not have AEU, and iii) single-exon genes. A supporting goal is the three-fold assessment of the approach that encompassed; 1) the use separate training and validation data sets, 2) gene set enrichment and gene functional analyses of the results, and 3) comparison of predicted and reported AS events.

## Methods

### Training data set

Survival, clinical and exon expression information from 250 patients diagnosed with GBM was obtained from The Cancer Genome Atlas repository, May 2011 data freeze (https://tcga-data.nci.nih.gov/tcga/). Surgical samples had a minimum of 80% tumor nuclei and maximum of 50% necrosis. The clinical or epidemiological variables considered in the analysis of exon expression included treatment (levels: chemo-radiation-targeted [CRT], chemo-radiation-non targeted [CRnT], radiation [R], other therapies [OTHER], and no therapy [NONE]); racial ethnicity (white Caucasian and others); and gender (male and female). These clinical factors were previously found to be associated with survival [9]. The survival indicator was the time from diagnosis to death, expressed in months.

Exon expression measurements from a frozen sample from each patient were obtained using the AffymetrixGeneChip® Human Exon 1.0 ST Array (Affymetrix, Santa Clara, CA). This platform includes information from 1,432,143 probe sets representing known and predicted exons on both strands of the genome that have been mapped to more than 25,000 genes. Intensity data was log-2 transformed and normalized using quantile and RMA normalization at the probe level following the procedures described in Beehive (http://stagbeetle.animal.uiuc.edu/Beehive). Probes sets within exons were collapsed using a Tukey biweight function that provides an iterative reweighed measure of central tendency. This robust statistic provides a single exon expression that is not heavily influenced by extreme probe expression levels [19].

### Model

Three specifications of this model accommodated three groups of genes: 1) multi-exon genes exhibiting AEU, 2) multi-exon genes with no evidence of AEU, and 3) single-exon genes. The first, second and third model specifications correspond to equations [[1]], [[2]] and [[3]], respectively. Within gene, a general exon expression mixed model was developed to describe the association between and exon expression and GBM survival adjusted for other clinical factors:

(1) $$y_{\text{ijklmn}}=µ+G_{i}+R_{j}+T_{k}+b_{2}S_{l}+X_{m}+b_{3m}{\left(\text{SX}\right)}_{\text{lm}}+P_{n}+e_{\text{ijklmn}}$$

(2) $$y_{\text{ijklmn}}=µ+G_{i}+R_{j}+T_{k}+b_{4}S_{l}+X_{m}+P_{n}+e_{\text{ijklmn}}$$

(3) $$y_{\text{ijklmn}}=µ+G_{i}+R_{j}+T_{k}+b_{5}S_{l}+P_{n}+e_{\text{ijklmn}}$$

where y_ijklmn_ denotes the expression of the m^th^ exon (X_m_), recorded on the n^th^ patient (P_n_) that has the i^th^ gender (G_i_), j^th^ race/ethnicity (R_j_), and received the k^th^ therapy (T_k_). Survival after diagnosis (S_l_), expressed in months, was fitted as a covariate. The parameters b_2_, b_4_, and b_5_ describe the overall change in expression per additional survival month across all exons, and b_3m_ descrbe the deviation in change in expression per additional survival month for the m^th^ exon in multi-exon genes that have AEU. In addition, e_ijklmn_ is the residual associated with the y_ijklmn_ observation, and SX denotes the interaction between survival and exon. In this model the fixed effects were; gender, race/ethnicity, therapy, and survival after diagnosis. The random effects exon, interaction between survival and exon, and patient were assumed to be independent and follow a Gaussian distribution with mean zero and its own variance.

A significant interaction between survival and exon effect constitutes evidence of an AEU scenario and, thus, differential survival across exons (group 1 genes). This model can be used to identify AS biomarkers of GBM survival that exhibit AEU. A significant survival after diagnosis effect together with a non-significant interaction between survival and exon effect constitutes evidence of a general association between gene expression and survival, regardless of exon (group 2 genes). This result can be used to identify multi-exon biomarkers of GBM survival that do not exhibit AEU.

The specification for the single-exon genes (group 3 genes) is a reduced version of the full multi-exon model that excludes exon and interaction between survival and exon. A significant survival effect is evidence of association between the single-exon gene expression and survival and can be used to identify single-exon biomarkers of GBM survival.

The hierarchical structure of the model used to identify AEU and gene expression associations with survival stems from the presence of two type of descriptive parameters: general or population-level and group-level parameters. There is a general or population-level association between exon expression and survival across all the exons of the gene, and group-level exon-specific deviations from the overall association that reveal alternative exon usage. A second hierarchical structure stems from a population-level exon expression, and patient-specific deviations from the overall expression level. The within-gene analysis supported a gene-centric strategy to uncover expression profiles associated with survival. The analysis of all exon information within a gene-allowed accounting for the covariance between exon expression within a gene and the hierarchical nature of the model allows the inclusion of the covariance between exon-expression within a patient. The analysis of expression data at the exon level permitted the identification of AEU by testing the null hypothesis of no differential association between expression and survival across exons within a gene.

False Discovery Rate adjustment (FDR) of the P-values allowed controlling for multiple testing [20]. In addition, a more stringent P-value threshold was considered for the detection of AEU associations with a significant interaction between survival and exon) than the main survival effect in the multi-exon scenarios. The more stringent P-values required for detection of AEU accounted for the multiple comparisons of the survival-expression associations among potentially numerous exons. A separate FDR adjustment of the P-values from the single-exon analysis was implemented because of the different number of parameters between the multi- and single-exon models. In this study, the significance threshold P-value < 5.0E-4 corresponds to a FDR-adjusted P-value < 0.05 or multi exon genes and to a FDR adjusted P-value < 0.1 for genes with single exon. The mixed effects model was evaluated in a restricted maximum-likelihood framework using the SAS 9.2 MIXED procedure (http://www.sas.com/).

Thus, three types of evidence were used to identify AEU: a) significant variations in the associations between exon expression and survival across a gene, b) consistent (over or under-expressed) differential expression in more than two consecutive exons, and c) a minimum exon differential expression (< 0.995 or > 1.005 fold change / additional survival month). Consistent patterns of expression across consecutive exons were identified using a moving average analysis [21]. A moving average analysis that computes the average expression across multiple exons at a time was used to predict a continuous trajectory of exon expression across the gene. This moving average trend of exon expression across the gene facilitated the identification of consistent changes in the pattern of over or under-expression across the exons within a gene.

Functional and pathway analyses of the genes exhibiting significant evidence (P-value < 5.0E-4) of AEU associated with GBM survival used hypergeometric tests and was implemented in DAVID [22,23]. Gene set enrichment analysis (GSEA) of the association between expression and GBM survival among all the genes studied in the platform followed the approach described by Subramanian et al. [24] implemented in BABELOMICS 4.3 [25]. For this analysis, the association between each gene and survival was characterized by the estimate of change in expression per additional survival month standardized by the standard error of the estimate. The enrichment of Gene Ontology (GO; http://www.geneontology.org/) biological processes, molecular functions, and KEGG (http://www.genome.jp/kegg/pathway.html) pathways was investigated. Finally, P-values of the enriched categories were adjusted for multiple testing using the FDR correction.

The exon expression estimates and the moving average trajectory of the estimates across individual genes were aligned to known or predicted alternative transcript variants reported in the AceView database (http://www.ncbi.nlm.nih.gov/IEB/Research/Acembly/) that are available through the UCSC Genome Browser (http://genome.ucsc.edu). This visualization strategy facilitated the interpretation of results and the AS models offered an independent *in silico* confirmation of the AEU events identified.

### Validation dataset

Genes exhibiting AEU in the training data set were confirmed in an independent set of 78 patients obtained from TCGA (May 2011 data freeze). The reliability of the exon expression profiles associated with survival identified in the training data set was assessed using a two-stage approach. First, the parameter estimates (i.e. changes in exon expression per one additional month of survival) that were obtained from the analysis of the training data set were applied to the covariate information from the patients in the validation data set, and predictions of exon expression were obtained. Second, the predicted exon expression values were compared to the corresponding observed expression values. The performance of the training estimates was evaluated using the coefficient of determination (R^2^) that represents the fraction of the total variation of the expression associated with survival, exon and the rest of the model terms [26]. High R^2^ on the validation data set based on the training data set estimates indicate the reliability of the exon expression patterns identified.

## Results and discussion

Expression measurements of 269,951 exons from 25,403 genes were analyzed. Of these, 2,857, 20,288, 1,965 and 293 genes had 1, 2 to 24, 25 to 49, and 50 or more exons, respectively. The number of exons per gene ranged from 1 to 191 and averaged 10.75 exons per gene. Table 1 summarizes the distribution of the 250 and 78 individuals diagnosed with GBM analyzed in the training and validation data sets respectively, across clinical factors, and survival descriptive statistics. The distribution of observations across clinical factors was consistent across data sets.

**Table 1:** Distribution of patients across clinical factors by data set

		**Training data set**		**Validation data set**	
		**Number**	**Percentage**	**Number**	**Percentage**
Patients		250	76.22	78	23.78
Race^1^	Caucasian	222	88.80	71	91.03
	Other	28	11.20	07	8.97
Gender	Females	94	37.60	29	37.18
	Males	156	62.40	49	62.82
Therapy^2^	R	63	25.20	21	26.92
	CRT	27	10.80	07	8.97
	CRnT	99	39.60	31	39.74
	OTHER	35	14.00	10	12.82
	NONE	26	10.40	09	11.54
Survival (months)		17.46	0.16 - 128	15.02	0.10 – 77.57

^1^Race: White Caucasian, and all other race-ethnicity groups.

^2^Therapy; R: radiation therapy alone; CRnT: chemotherapy plus radiation and no targeted therapy; CRT: chemotherapy plus radiation and targeted therapy; OTHER: all other therapies; None: no therapy.

### Multi-exon genes exhibiting exon-dependent association with glioblastoma multiforme survival

At a FDR-adjusted P-value < 0.05 (approximately equivalent to an unadjusted P-value < 5.0E-4) threshold, 2477 multi-exon genes exhibited AEU associated with survival (group 1 genes), 24 multi-exon genes exhibited expression associated with survival albeit no evidence of AEU (group 2 genes), and 8 single-exon genes exhibited expression associated with survival (group 3 genes). A similar number (1,478) of differentially expressed genes was associated short- versus long-term glioblastoma survival [27]. The higher number of associations detected in the present study could be attributed to the analysis of exon-level profiles instead of gene-level profiles. At unadjusted P-value < 1.0E-5, P-value < 1.0E-6 (approximately equivalent to a FDR-adjusted P-value < 1.0E-2), P-value < 1.0E-7, P-value < 1.0E-8 (approximately equivalent to a FDR-adjusted P-value < 5.0E-4), the number of genes exhibiting AEU (group 1 genes) were 592, 313, 201, and 129 respectively.

Table 2 summarizes the 36 multi-exon genes that have the most significant (P-value < 0.11) AEU or exon-dependent association with GBM survival (group 1 genes). Additional file 1: Table S1 lists the results for the 129 multi-exon genes that exhibit evidence of AEU at P-value < 1.0E-8.

**Table 2:** Top 36 multi-exon genes that have significant alternative exon usage associated with glioblastoma multiforme survival

**Gene Symbol**	**Estimate**^**1**^	**SE**^**2**^	**P-value AEU**^**3**^	**Fold change**^**4**^	**Exon Count**^**5**^	**Literature**^**6**^
*Ttn*	0.0007	0.0001	4.2E-38	0.9993	340	[28]^G^
*Smg1*	0.0017	0.0002	2.0E-24	1.0001	209	[29]^AS^
*Neb*	0.0007	0.0001	3.2E-21	0.9973	180	[30,31]^C, AS^
*Pkd1*	0.0010	0.0001	2.0E-19	1.0018	163	[32]^G, AS^
*Herc2p2*	0.0008	0.0001	2.3E-19	1.0012	163	NA
*Syne1*	0.0018	0.0002	3.0E-18	0.9984	152	[9]^G^
*Snrpn*	0.0018	0.0002	3.8E-18	1.0020	151	[33,34]^G, AS^
*Pde4dip*	0.0016	0.0002	1.3E-17	0.9993	146	[35,36]^G, AS^
*Golga8c*	0.0031	0.0004	4.2E-17	1.0005	141	NA
*Sspo*	0.0009	0.0001	1.2E-16	1.0003	137	NA
*Ankrd36*	0.0026	0.0003	1.3E-16	1.0018	137	NA
*Tbc1d3*	0.0008	0.0001	2.4E-16	1.0026	135	[37]^C^
*Flj45340*	0.0018	0.0002	5.5E-16	1.0007	131	NA
*Anapc1*	0.0009	0.0001	5.8E-15	0.9990	122	[38,39]^C, AS^
*Syne2*	0.0012	0.0002	6.2E-15	1.0017	115	[40]^C, AS^
*Nbpf10*	0.0035	0.0005	1.3E-14	0.9992	118	[41]^C, AS^
*Muc19*	0.0015	0.0002	1.4E-14	1.0001	118	[42]^C, AS^
*Obscn*	0.0006	0.0001	1.5E-14	0.9999	118	[28,43]^G, AS^
*Npipl3*	0.0019	0.0003	4.1E-14	1.0014	114	NA
*Dst*	0.0013	0.0002	9.4E-14	0.9997	111	[44,45]^G, AS^
*Col7a1*	0.0011	0.0001	1.4E-13	1.0001	109	[46,47]^C, AS^
*Ubr4*	0.0011	0.0001	1.4E-13	0.9994	109	[48,49]^C, AS^
*Hmcn1*	0.0006	0.0001	2.0E-13	0.9975	109	[50]^C, AS^
*Ryr2*	0.0011	0.0001	2.7E-13	0.9974	107	[51,52]^G, AS^
*Macf1*	0.0011	0.0002	3.1E-13	0.9975	106	[53,54]^G, AS^
*Mdn1*	0.0006	0.0001	3.5E-13	0.9993	106	NA
*Col4a5*	0.0008	0.0001	3.5E-13	0.9992	106	[55,56]^C, AS^
*Ryr1*	0.0007	0.0001	4.2E-13	0.9998	105	[31,57,58]^G, AS^
*Golga6l5*	0.0013	0.0002	5.2E-13	1.0021	104	NA
*Ryr3*	0.0009	0.0001	1.3E-12	0.9962	102	[59]^C, AS^
*Dnah14*	0.0007	0.0001	2.0E-12	0.9990	99	NA
*Herc2*	0.0006	0.0001	3.1E-12	1.0003	97	[60]^AS^
*Dnah8*	0.0005	0.0001	4.7E-12	0.9997	96	NA
*Nomo1*	0.0007	0.0001	4.9E-12	0.9996	95	NA
*Gpr98*	0.0016	0.0002	5.9E-12	0.9948	95	[61]^C, AS^
*Golga6a*	0.0017	0.0002	7.8E-12	1.0009	93	NA

^1^Estimate: exon-survival interaction variance indicator of alternative exon usage.

^2^SE: standard error of the estimate.

^3^P-value AEU: unadjusted P-value of alternative exon usage or exon-dependent association between expression and glioblastoma multiforme survival.

^**4**^Fold change: fold change in average exon expression per additional survival month.

^5^Exon Count: number of exons in the gene.

^**6**^Literature: review of studies that reported associations of the gene with cancers:

^G^: reported association of gene with glioblastoma multiforme,

^C^: reported association of gene with cancer other than glioblastoma multiforme,

^AS^: identification of different variants due to alternative splicing (AS) event,

NA: not available.

The nature of the association between expression and GBM was characterized by the sign and value of the expression change per additional month in survival. Tables 2, 3 and 4 include a general, exon-independent, gene-wise estimate of expression fold change per additional survival month for completeness. The meaning of this fold change estimate is straightforward for genes in groups 2 and 3 because these genes exhibit a single, general, and exon-independent association with GBM survival. The general fold change estimate for group 1 genes must be considered in the context that these genes have an exon-dependent association with GBM.

**Table 3:** Ten most significant KEGG and GO categories enriched among the genes displaying alternative exon usage

**Source**	**Category**	**Gene Count**^1^	**FDR P-value**^2^
**KEGG Pathway**	(hsa04510) focal adhesion	86	3.2E-21
	(hsa04512) ecm-receptor interaction	51	8.5E-20
	(hsa02010) abc transporters	30	2.5E-12
	(hsa04810) regulation of actin cytoskeleton	66	1.7E-07
	(hsa05412) arrhythmogenic right ventricular cardiomyopathy	32	5.9E-06
	(hsa05414) dilated cardiomyopathy	37	1.3E-06
	(hsa04070) phosphatidylinositol signaling system	31	1.2E-05
	(hsa05222) small cell lung cancer	31	3.6E-04
	(hsa05410) hypertrophic cardiomyopathy	32	1.3E-04
	(hsa05200) pathways in cancer	73	3.0E-02
**GO Biological Process**	(GO:0051056) regulation of small GTPase mediated signal transduction	105	0.055
	(GO:0022610) biological adhesion	197	2.7E-22
	(GO:0007155) cell adhesion	197	2.3E-22
	(GO:0046578) regulation of Ras protein signal transduction	79	5.0E-15
	(GO:0035023) regulation of Rho protein signal transduction	51	1.7E-15
	(GO:0007010) cytoskeleton organization	129	1.3E-15
	(GO:0030029) actin filament-based process	85	2.3E-14
	(GO:0007018) microtubule-based movement	51	2.1E-12
	(GO:0016568) chromatin modification	89	1.9E-12
	(GO:0051276) chromosome organization	132	1.4E-12
**GO Molecular Function**	(GO:0030554) adenyl nucleotide binding	451	9.9E-59
	(GO:0005524) ATP binding	433	2.2E-59
	(GO:0032559) adenyl ribonucleotide binding	437	2.0E-59
	(GO:0001882) nucleoside binding	456	6.3E-58
	(GO:0001883) purine nucleoside binding	451	1.5E-56
	(GO:0017076) purine nucleotide binding	480	5.2E-44
	(GO:0032555) purine ribonucleotide binding	466	2.9E-44
	(GO:0032553) ribonucleotide binding	466	2.9E-44
	(GO:0000166) nucleotide binding	523	7.4E-39
	(GO:0003774) motor activity	86	1.3E-34

^1^Gene Count: number of genes that have significant alternative exon usage within category.

^2^FDR-adjusted P-value: False discovery rate adjusted P-value of the hypergeometric test of category enrichment.

**Table 4:** Top 5 multi-exon genes that have significant exon-independent association with glioblastoma multiforme survival

**Gene Symbol**	**Estimate**^**1**^	**SE**^**2**^	**Fold Change**^**3**^	**P-value**^**4**^	**P-value AEU**^**5**^	**Exon Count**^**6**^	**Literature**^**7**^
*Sirt2*	0.0337	0.0092	1.0236	3.2E-04	2.5E-03	17	[62]^G^
*Six1*	0.0056	0.0015	1.0039	3.3E-04	2.7E-01	5	[63]^C^
*Loc100289627*	0.0079	0.0022	1.0055	3.8E-04	4.3E-01	2	NA
*Sema3e*	−0.0256	0.0066	0.9824	1.3E-04	2.4E-03	18	[64]^C^
*Golga8j*	−0.0536	0.0141	0.9635	1.7E-04	1.1E-03	20	[65]^C^

^1^Estimate: change in average exon expression per additional survival month (in log2 units).

^**2**^SE: standard error of the estimate.

^**3**^Fold change: fold change in average exon expression per additional survival month.

^**4**^P-value: unadjusted P-value of the change in average exon expression per additional survival month.

^**5**^P-value AEU: non-significant (P-value > 1.0E-03) evidence of alternative exon usage.

^**6**^Exon Count: number of exons in the gene.

^**7**^Literature: review of studies that reported associations of the gene with cancers:

^G^: reported association of gene with glioblastoma multiforme,

^C^: reported association of gene with cancer other than glioblastoma multiforme,

NA: not available.

The top 36 genes exhibiting significant evidence of AEU had a minimum of 90 exons (Table 2). This result suggests that genes with high number of exons are more likely to experience AEU events that influence GBM survival than genes with few exons. It is unlikely that high number of exons biased the identification of AEU due to the stringent P-value threshold used.

Most of the 36 genes that had significant AEU association with GBM survival have been associated with cancer. Relevant literature references are summarized in Table 2. There were 10 genes including titin (*Ttn*), polycystic kidney disease 1 (*Pkd1*), spectrin repeat containing, nuclear envelope 1 (*Syne1*), small nuclear ribinucleoprotein (*Snrpn*), phosphodiesterase 4D interacting protein (*Pde4dip*), obscurin (*Obscn*), dystonin (*Dst*), microtubule-actin cross-linking factor 1 (*Macf1*), ryanodine receptor 1 (*Ryr1*), and ryanodine receptor 2 (*Ryr2*), that had been previously associated with GBM. Additionally, 13 genes have been previously associated to cancers other than GBM including; Smg-1 homolog (*Smg1*), Nebulin (*Neb*), TBC1 domain family, member 3 (*Tbc1d3*), Anaphase promoting complex subunit 1 (*Anapc1*), Spectrin repeat containing, nuclear envelope 1 (*Syne2*), Neuroblastoma breakpoint family, member 10 (*Nbpf10*), Mucin 19 (*Muc19*), Collagen, type VII, alpha 1 (*Col7a1*), Ubiquitin protein ligase E3 component n-recognin 4 (*Ubr4*), Hemicentin 1 (*Hmcn1*), Collagen, type IV, alpha 5 (*Col4a5*), Ryanodine receptor 3 (*Ryr3*), and G protein-coupled receptor 98 (*Gpr98*).

Previous reports confirmed the overall relationship between the top genes exhibiting AEU and GBM identified in this study. Reports on the overall relationship between genes and GBM are hereby reviewed since the AEU pattern for these genes has not been previously described. The TTN protein is encoded by *Ttn*, and is responsible for the passive elasticity of cells. A mutation resulting in an altered TTN was associated with GBM [28]. *Pkd1* was over-expressed during the progression of low-grade to high-grade gliomas [32]. *Syne1* has been associated with increased GBM survival [9]. Under-expression of *Snrnp* was observed in older GBM patients compared to younger patients [33]. *Pde4dip* is down-regulated in glioma cell lines treated with dB-cAMP that reduces the invasiveness, proliferation and migratory properties of glioma cells and increases the survival of glioma cells lines compared to untreated cell lines [35]. The mutation R4558H in *Obscn* has been associated with GBM [28]. Likewise, a mutation in *Dst* that indirectly regulates the expression of *Otub1* (through regulation of mir-15b has been associated with GBM [44]. Reduced expression of *Macf1* has been observed in glioma cells treated with IL-13 cytotoxin that causes the cells to undergo necrosis. Thus, down-regulation of the expression of *Macf1* was associated with increased GBM survival [53]. *Ryr1* was under-expressed in high-grade gliomas relative to primary (low-grade) gliomas [57]. On the other hand, *Ryr2* was over-expressed in invasive GBM cells compared to normal control cells [51].

### Functional and pathway analyses of the multi-exon genes exhibiting exon-independent association with glioblastoma multiforme survival

The 2,477 genes exhibiting significant evidence of AEU associated with GBM survival were further investigated using functional and pathway analyses. At FDR-adjusted P-value < 0.05, 15 KEGG pathways, 87 GO biological processes, and 70 GO molecular functions were enriched. The top 10 pathways, biological processes and molecular functions are summarized in Table 3. Additional file 1: Table S2 lists all KEGG pathways and GO categories with FDR-adjusted P-value < 0.05. Among the 15 pathways significantly enriched, focal adhesion was the most significant pathway encompassing 86 genes. This result was consistent with many reports of the critical role of focal adhesion and gliomas [66-68]. The enrichment of the extra-cellular matrix- (ECM-) receptor interaction pathway detected in this study has been reported in other cancers [69,70]. The ATP-binding cassette (ABC) transporter pathway has been associated with gliomas [71]. Our finding of small cell lung carcinoma pathways enrichment associated with GBM was consistent with the multiple studies that have identified commonalities among these cancers [72]. The most enriched biological process among the AEU genes associated with GBM survival included regulation of small GTPase mediated signal transduction (RSGST), and neuron development that has been associated with neuroblastoma [73]. The enrichment of biological adhesion confirms our focal adhesion results. Among the top 70 GO molecular functions significantly enriched were adenyl ribonucleotide binding, ATP binding, nucleotide binding and helicase activity. These related nucleotide binding functions have been associated with GBM [74].

### Multi-exon genes exhibiting exon-independent association with glioblastoma multiforme survival

At unadjusted P-value < 5.0E-4, 24 multi-exon genes exhibited exon-independent association with GBM survival (group 2 genes). In other words, there was no evidence of AEU in these genes because the expressions of all the exons were consistently associated with GBM survival and a single general or overall association between the gene and survival can be identified. Table 4 lists the top five multi-exon genes that have the most significant exon-independent association with GBM survival. Additional file 1: Table S3 lists the results for the 24 multi-exon genes exhibiting expression associated with survival albeit no evidence of AEU at P-value < 5.0E-4.

Among the 24 multi-exon genes that were associated with GBM survival on a general, exon-independent manner, the five genes that have the lower AEU evidence (AEU unadjusted P-value > 1.0E-3, approximately FDR adjusted P-value > 0.1) are listed in Table 4. The expression of three of these genes increased with increasing survival. Noteworthy was the low number of exons in these genes, relative to the higher number of exons in genes exhibiting evidence of AEU.

Four of five multi-exon genes have been associated to different cancers in studies listed in Table 4, and the remaining gene (LOC100289627) is similar to Guanine nucleotide-binding protein subunit beta-2-like 1. Sirtuin2 (*Sirt2*) has been associated with GBM while the other three genes golgin subfamily A member 8J (*Golga8j*), semaphorin 3E (*Sema3e*) and SIX homeobox 1 (*SIX1*) were associated with other cancers. Under-expression of *Sirt2* has been reported in glioma cells relative to control cells [62]. This result is also consistent with our findings that higher levels of *Sirt2* were associated with higher GBM survival *Golga8j* has been associated with pancreatic cancer and the trend is consistent with our finding of lower GBM survival with higher expression levels of this gene [65]. *Sema3e* promotes invasiveness and metastatic ability of the cancerous cells [64]. *Sema3e* is associated with many cancers like prostate cancer colon cancer and lung adenocarcinoma [75]. This result is consistent with our findings that higher levels of *Sema3e* were associated with lower GBM survival. The gene *Six1* is associated with lower survival in cancerous cells [63]. This result is inconsistent with our results showing an increase in *Six1* expression associated with an increase in GBM survival.

### Single-exon genes associated with glioblastoma multiforme survival

Eight single-exon genes were associated with GBM survival (group 3 genes) at unadjusted P-value < 5.0E-4 (Table 5). Among these, three genes had a negative relationship such that lower expression levels were associated with higher survival. Four members of the family of small nucleolar RNA CD box (*Snord*) genes were associated with GBM survival, and three had a positive association such that higher expression levels were associated with higher survival. *Snord* are a type of small nucleolar RNA (SnoRNA) that guides the methylation of rRNAs and snRNAs. These snoRNAs can target other RNAs and are associated with carcinogenesis. Reduced and dysregulated expression of snoRNAs have been associated with progression of many human malignancies [78]. Along with their loss in brain tumorigenesis, snoRNAs have been also linked to other cancers such as prostate, breast and lung cancer [76,78]. In this study, a positive association between the levels of H1 histone family member 0 (*H1f0*) and GBM survival was identified. The expression of *H1f0* was high in breast tumor cells, and decreased when the breast tumor cell lines were reverted-back into normal ME carcinoma cells [77].

**Table 5:** Single-exon genes associated with glioblastoma multiforme survival

**Gene symbol**	**Estimate**^**1**^	**SE**^**2**^	**Fold Change**^**3**^	**P-value**^**4**^	**Literature**^**5**^
*Hist1h1t*	0.0118	0.0024	1.0082	2.5E-06	NA
*Snord116*-*11*	0.0101	0.0025	1.0070	9.7E-05	[76]^C^
*Loc729852*	−0.0074	0.0018	0.9949	5.8E-05	NA
*Snord123*	−0.0087	0.0025	0.9940	4.8E-04	[76]^C^
*Snord104*	0.0067	0.0019	1.0047	4.1E-04	[76]^C^
*Dkfzp779l1853*	−0.0083	0.0023	0.9943	3.9E-04	NA
*H1f0*	0.0062	0.0017	1.0043	2.3E-04	[77]^C^
*Snord28*	0.0166	0.0044	1.0116	1.8E-04	[76]^C^

^1^Estimate: change in gene expression per additional survival month (in log2 units).

^2^SE: standard error of the estimate.

^3^Fold change: fold change in gene expression per additional survival month.

^4^P-value: unadjusted P-value of the change in average exon expression per additional survival month;

^5^Literature: review of studies that reported associations of the gene with cancers:

^C^: reported association of gene with cancers other than glioblastoma multiforme,

NA: not available.

### Gene set enrichment analyses of all genes in consideration of their association with glioblastoma multiforme survival

Gene set enrichment analysis considered the level and sign of association between the expression of all the genes studied and GBM survival. At FDR-adjusted P-value < 0.05, 94 KEGG pathways, 402 GO biological processes, and 203 GO molecular functions were enriched. Results from the top 10 most significant pathways, biological processes and molecular functions are summarized in Tables 6, 7 and 8. Additional file 1: Table S4, S5 and S6 lists all biological processes, molecular functions and pathways respectively that have FDR-adjusted P-value < 0.05. Pathways and GO categories are characterized in GSEA by the number of genes that have a positive or negative association between expression and GBM survival, by the log odds ratio indicating whether the category is more enriched among the genes that have a positive or negative association and the corresponding P-value.

**Table 6:** Ten most significant GO biological processes from the gene set enrichment analysis of the genome

**GO Identifier**	**GO Biological Process**	**Over-Expressed Gene**^**1**^	**Under-Expressed Genes**^**2**^	**Log Odds Ratio**^**3**^	**FDR P-value**^**4**^
GO:0046907	intracellular transport	357	560	−0.7338	3.79E-24
GO:0034613	cellular protein localization	245	433	−0.8490	4.78E-24
GO:0043067	regulation of programmed cell death	351	490	−0.6110	1.68E-15
GO:0016192	vesicle-mediated transport	271	400	−0.6639	1.16E-14
GO:0006629	lipid metabolic process	424	538	−0.5148	1.30E-12
GO:0044265	cellular macromolecule catabolic process	373	485	−0.5379	2.10E-12
GO:0044255	cellular lipid metabolic process	346	457	−0.5528	2.41E-12
GO:0050793	regulation of developmental process	442	549	−0.4932	4.27E-12
GO:0007049	cell cycle	418	522	−0.4978	1.11E-11
GO:0009966	regulation of signal transduction	414	509	−0.4812	9.93E-11

^1^Over-Expressed Genes: number of genes that have a positive association between expression and glioblastoma multiforme survival.

^2^Under-Expressed Genes: number of genes that have a negative association between expression and glioblastoma multiforme survival.

^3^Log Odds Ratio: indicates whether the category is more enriched among the genes that have a positive association between expression and survival relative to the enrichment among the genes that have a negative association between expression and glioblastoma survival (positive log_e_ odds ratio) or vice versa (negative log_e_ odds ratio). Extreme values indicate higher difference in the enrichment percentages between the positive and negative association groups meanwhile values close to zero indicate similar enrichment percentages between positive and negative association groups.

^4^FDR-adjusted P-value: False discovery rate adjusted P-value of the log odds ratio test.

**Table 7:** Ten most significant GO molecular functions from the gene set enrichment analysis

**GO Identifier**	**GO Molecular Function**	**Over-Expressed Gene**^**1**^	**Under-Expressed Genes**^**2**^	**Log Odds Ratio**^**3**^	**FDR P-value**^**4**^
GO:0000287	magnesium ion binding	196	300	−0.6962	3.23E-11
GO:0016818	hydrolase activity, acting on acid anhydrides, in phosphorus containing anhydrides	419	521	−0.4933	5.21E-11
GO:0016462	pyrophosphatase activity	417	520	−0.4962	5.21E-11
GO:0016817	hydrolase activity, acting on acid anhydrides	428	527	−0.4834	9.62E-11
GO:0016773	phosphotransferase activity, alcohol group as acceptor	393	475	−0.4624	5.64E-09
GO:0016301	kinase activity	421	501	−0.4473	5.64E-09
GO:0016788	hydrolase activity, acting on ester bonds	349	429	−0.4781	9.84E-09
GO:0003723	RNA binding	357	437	−0.4741	9.84E-09
GO:0030695	GTPase regulator activity	193	260	−0.5651	4.10E-07
GO:0016874	ligase activity	205	272	−0.5501	4.10E-07

^1^Over-Expressed Genes: number of genes that have a positive association between expression and glioblastoma multiforme survival.

^2^Under-Expressed Genes: number of genes that have a negative association between expression and glioblastoma multiforme survival.

^3^Log Odds Ratio: indicates whether the category is more enriched among the genes that have a positive association between expression and survival relative to the enrichment among the genes that have a negative association between expression and glioblastoma survival (positive log_e_ odds ratio) or vice versa (negative log_e_ odds ratio). Extreme values indicate higher difference in the enrichment percentages between the positive and negative association groups meanwhile values close to zero indicate similar enrichment percentages between positive and negative association groups.

^4^FDR-adjusted P-value: False discovery rate adjusted P-value of the log odds ratio test.

**Table 8:** Ten most significant KEGG pathways from the gene set enrichment analysis of the genome

**KEGG Identifier**	**KEGG Pathway**	**Over-Expressed Gene**^**1**^	**Under-Expressed Genes**^**2**^	**Log Odds Ratio**^**3**^	**FDR P-value**^**4**^
hsa03010	ribosome	119	16	−2.4779	9.7E-10
hsa00010	glycolysis / gluconeogenesis	57	27	−1.1614	3.6E-04
hsa00190	oxidative phosphorylation	103	39	−0.9392	3.6E-04
hsa05212	pancreatic cancer	54	45	−0.9460	4.7E-04
hsa05130	pathogenic escherichia coli infection	44	41	−1.0575	4.7E-04
hsa00240	pyrimidine metabolism	42	78	−0.8800	5.0E-04
hsa03050	proteasome	33	32	−1.0965	7.2E-04
hsa00280	valine, leucine and isoleucine degradation	20	48	−1.1353	8.5E-04
hsa04662	b cell receptor signaling pathway	34	65	−0.9084	8.5E-04
hsa05223	non-small cell lung cancer	25	52	−0.9922	9.0E-04

^1^Over-Expressed Genes: number of genes that have a positive association between expression and glioblastoma multiforme survival.

^2^Under-Expressed Genes: number of genes that have a negative association between expression and glioblastoma multiforme survival.

^3^Log Odds Ratio: indicates whether the category is more enriched among the genes that have a positive association between expression and survival relative to the enrichment among the genes that have a negative association between expression and glioblastoma survival (positive log_e_ odds ratio) or vice versa (negative log_e_ odds ratio). Extreme values indicate higher difference in the enrichment percentages between the positive and negative association groups meanwhile values close to zero indicate similar enrichment percentages between positive and negative association groups.

^4^FDR-adjusted P-value: False discovery rate adjusted P-value of the log odds ratio test.

Noteworthy was that all top ten results had negative log odds ratio indicating that the categories were more enriched among the genes that have a negative association between expression and survival relative to the enrichment among the genes that have a positive association between expression and GBM survival. Negative log_e_ odds ratio indicates that the enrichment was higher among the genes with negative association with GBM survival. Positive log odds ratios were observed for less significant (P-value < 0.05) pathways and categories. The more extreme log odds ratios observed in the GSEA of KEGG pathways indicate higher difference between the enrichment percentages in the positive and negative association groups meanwhile values close to zero in the GSEA of GO categories indicate lower differences in the enrichment percentages between positive and negative association groups.

Among the most differentially enriched pathways (Table 6) were the pancreatic and non-small cell lung cancer pathways. Additional pathways identified in this study that have been associated with gliomas include glycolysis/gluconeogenesis [79] and oxidative phosphorylation [80]. Among the top enriched GO biological processes, lipid metabolism and cell cycle have been associated with glioma [81,82]. Likewise, the GO molecular functions of hydrolase and ligase activity have been linked to glioma [83,84].

### Demonstration of alternative exon usage

**Figure 1:**
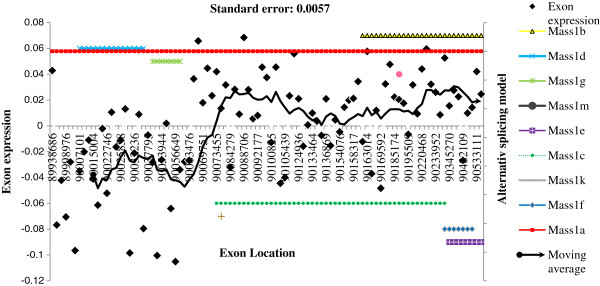
***Gpr98 *exon expression, moving average, and alternative splicing models. ***Gpr98*: G-protein coupled receptor 98. X-axis: location in the gene (in bp). Left Y-axis: change in exon expression per additional survival month calculated from the alternative exon usage model. Full diamond black markers: exon expression from the alternative exon usage model (Exon expression). Continuous black line: moving average pattern of expression based on 10 exons. Standard Error: standard error of the exon expression estimate. Right Y-axis: indicator of AceView alternative splicing model. Colored continuous and dotted lines including cross, triangle, square, circle, line, and plus markers: indicator of the location of the AceView alternative splicing models (AceView models indicate exon series or cassette locations in the gene).

**Figure 2:**
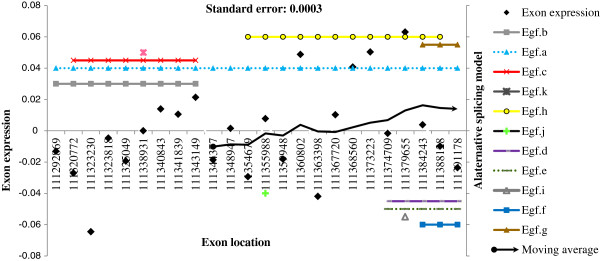
***Egf *exon expression, moving average, and alternative splicing models. ***Egf*: epidermal growth factor. X-axis: location in the gene (in bp). Y-axis (left): change in exon expression per additional survival month calculated from the alternative exon usage model. Full diamond black markers: exon expression from the alternative exon usage model (Exon expression). Continuous black line: moving average pattern of expression based on 10 exons. Standard Error: standard error of the exon expression estimate. Right Y-axis: indicator of AceView alternative splicing model. Colored continuous and dotted lines including cross, triangle, square, circle, line, and plus markers: indicator of the location of the AceView alternative splicing models (AceView models indicate exon series or cassette locations in the gene).

The identification of patterns of differential exon expression across a gene and comparison against predicted AS models helped to confirm associations between AS and survival. Figures 1 and 2 depict patterns of exon expression associated with GBM survival and reported AS gene models for two genes that exhibited significant AEU associated with GBM survival. The patterns of two other genes that exhibited AEU are presented in Additional file 2: Figures S1 and S2. The two genes depicted in Figures 1 and 2 are G-protein coupled receptor 98 (*Gpr98*) and epidermal growth factor (*Egf*), respectively: The two genes depicted in the Additional file 2: Figures S1 and S2 are anaphase promoting complex subunit 1 (*Anapc1*) and HECT domain and RLD domain containing E3 ubiquitin protein ligase 2 (*Herc2*), respectively. The parallel alignment of estimated exon expression resulting from our analysis, the moving average trend, and the AS prediction from AceView offered *in silico* verification of the identified AEU [2]. The AS models are denoted by lines parallel to the x-axis and identify the corresponding exons. However, no expression values should be assigned to the AS model lines and experimental confirmation of the AEU cases identified in this study is necessary.

*Gpr98* is located on chromosome 5 and is highly expressed in the central nervous system (CNS) [85]. This gene has been associated with Usher syndrome characterized by hearing loss and progressive vision loss and Familial Febrile seizures [86,87]. *Gpr98* has been linked to cancer [88] and smaller variants of *Gpr98*, produced due to AS, have been associated with increased survival against lymphoblastic leukemia [61]. *Gpr98* exhibited AEU in this study and the expression of approximately 30 exons (out of 90 exons) exhibited significant association with GBM survival (Figure 1). Several over-expressed exons detected by our model are consistent with AS gene models including Mass1b, Mass1f, Mass1e, and Mass1c. Conversely, some under-expressed exons identified in our study are supported by gene models including Mass1d and Mass1g. These results are consistent with previous studies that indicated association of smaller transcripts of *Gpr98* with cancer survival by inducing apoptosis in cancerous cells [61]. In agreement with our GO analyses, *Gpr98* is affiliated to the enriched GO biological processes of cell adhesion, neuron development and sensory perception of mechanical stimulus. Additionally, *Gpr98* has the GO molecular function of cytoskeletal protein binding and ion binding. For *Gpr98*, the relative difference in R^2^ between the training and validation data sets was 18.1%. The Pearson correlation of the exon-survival associations between the training and validation data sets was 83.2%.

*Egf* is located on HAS 4 and over-expression of *Egf* has been associated with tumor progression and lower GBM survival [89]. *Egf* exhibited AEU and of the 24 exons analyzed, nine exons had significant associations with GBM survival. Several over-expressed exons detected in our analysis correspond to AS gene models including jdec03 and hdec03 (Figure 2). In accord with the pathway and functional analyses, *Egf* is part of many enriched KEGG pathways including focal adhesion, regulation of actin cytoskeleton, and cancer pathways. For *Egf*, the relative difference in R^2^ between the training and validation data sets was 24.9%. The Pearson correlation of the exon-survival associations between the training and validation data sets was 76.6%.

### Validation

The R^2^ is the percentage of the variation of the observations explained by the exon-based, gene-centric approach. Simply put, the R^2^ is an indicator of the correlation between exon expression and patient survival, adjusting for therapy, ethnicity, gender, among all terms accounted for in our model). The median R^2^ in the training and validation data sets were 75.7% and 63.4% for the multi- and single-exon genes significantly associated with survival. The relative difference in R^2^ between the training and validation data sets was 16.2%. The small drop in median R^2^ between the training and validating data set is a first, global indicator of the similar exon-survival relationship identified in both independent data sets. A difference between training and validating data is expected due to simple sampling effects such as between-subject variation.

Additional insight into the validation of associations detected in the training data sets was gained from the study of the correlation of the exons-survival association (e.g. estimated solution or slope) between training and validation data sets. For the multi- and single-exon genes significantly associated with survival, the median Pearson and Spearman correlations of the exon-survival associations between the training and validation data sets were 89.7% and 85.9%, respectively. The high correlation of the exon-survival associations relative to the drop in R^2^ model fit between training and validation data sets suggests that the exon associations with survival detected are more consistent or have lower noise than the other model terms including race, gender and therapy.

## Conclusions

In conclusion, AEU is a complex process and, thus, the detection and characterization of AEU associated with survival is challenging. The hierarchical model developed in this study allowed the simultaneous detection of differential expression of exons within a gene and differentially expressed genes associated with survival. From a total of 25,403 genes investigated, 2,477 multi-exon and 13 single exon genes were associated with GBM. Most of the significant genes detected by the model have been previously associated to GBM (27.78%) or other types of cancer (36.11%). This suggests that differential expression associated with AEU could be used as biomarkers for GBM and potentially other cancers. The AEU events detected for several genes (*Egf*, *Herc2*, *Gpr98* and *Anapc1*) were consistent with AS models in AceView.

The approach used to identify alternative exon usage and gene expression associated with survival adjusted for race, gender, and therapy differences among the patients analyzed. Thus, prognostic biomarkers of glioblastoma survival were identified. Stratified analyses of the patients by age, race, therapy, and gender or evaluation of potential interactions between exons and clinical factors could uncover predictive biomarkers and offer additional insights into the alternative exon usage associations that can lead to more personalized treatments and predictive tools.

Extensions to the hierarchical model proposed in this study to identify AEU can be considered. First, the model can incorporate information of the mapping of the exons to the gene. In addition, the distance between the exons can be accommodated on the variance-covariance matrix. This would allow modeling of potentially higher dependencies between proximal exons relative to distant exons. Second, the model can incorporate information on different splicing scenarios [2]. The hierarchical model can be applied to other cancer types and to indicators other than survival.

In this study, the vast majority of the exons within a gene mapped to one strand and few exons mapped to the other strand. Thus, AEU was studied among the exons that mapped to the most frequent strand. When sufficient information on both strands within a gene is available, our model allows the consideration of information across strands. This model would allow the study of sense-antisense gene overlap and its impact on AS and regulation of gene expression following the work of Sorana Morrissy et al [90]. Their work suggested an antisense transcription-mediated mechanism of splicing regulation in human cells.

A simple yet comprehensive analytical strategy for *in silico* identification of survival-associated alternative exon usage and general gene expression was demonstrated. The findings from this strategy and stringent biological and statistical thresholds were validated on an independent group of patients. Our approach can be used as a first step in the identification of cancer molecular biomarkers. A subsequent step is the experimental validation of the identified alternative exon usage and patterns of association between exons series or cassettes and survival. Experimental confirmation can be obtained from exon expression studies of the proliferation and survival of glioblastoma cell lines [91] or from studies of primary glioblastoma sphere cultures (gliomaspheres), an established in vitro model for cancer stem cell expansion [92]. Furthermore, the proposed analytical strategy can be applied to next-generation sequencing data, allowing a thorough investigation of the expression pattern associated with cancer survival and other complex phenotypes.

Further validation of alternative exon usage biomarkers can be carried out using Reverse Transcriptase-quantitative Polymerase Chain Reaction (RT-qPCR) assays or RNA-Seq technologies as new samples become available. Confirmation of results using RNA-Seq offers various advantages. Unlike exon arrays that require probe design and annotations, RNA-Seq can detect both known and previously unreported alternative splicing events and yet to be annotated transcripts. RNA-Seq has substantially better coverage for differentially expressed genes compared to arrays. The enhanced exon coverage and increased sensitivity to detect alternative splicing sites and differentially expressed exons constitutes a robust tool that can substantially enhance the understanding of alternative exon usage associated with complex phenotypes. The clinical benefit of alternative exon usage and associated exon cassettes or transcripts will be most valuable in cases when these biomarkers provide a significant improvement in the precision to predict survival over routinely available clinical tests and overall gene expression-based biomarkers. As a result, the impact of the superior biomarkers will likely be greatest in diseases with short average post-diagnostic survival such as glioblastoma multiforme. Likewise, alternative exon usage-based biomarkers have the potential to be helpful to predict phenotypes not accurately predicted by general gene-expression profiles. For several cancer types the recurrence of metastasis is the most compelling assessment of the efficacy of therapy. In these cases, accurate and replicable exon-based prognostic tools offer the most advantage and can complement available clinical tests.

## Competing interests

The authors declare that they have no competing interests.

## Authors’ contributions

AS obtained the exon expression data set, compiled information, performed the statistical analysis contributed to the interpretation of results, and drafted the manuscript. NVLS developed the clinical classifications, developed statistical routines, and contributed to the interpretation of the results. BRS wrote code to implement the statistical model and contributed to the manuscript. KRD contributed with the analysis. SRZ obtained funding for the study, participated in its conception, coordination, interpretation of results, and helped write the manuscript. All authors have read and approved the final version of this manuscript.

## Pre-publication history

The pre-publication history for this paper can be accessed here:

http://www.biomedcentral.com/1755-8794/5/59/prepub

## Supplementary Material

Additional file 1 **Table S1.** Lists the results for the 129 multi-exon genes that exhibit evidence of AEU at P-value < 1.0E-8. Lists the results for the 129 multi-exon genes that exhibit evidence of AEU at P-value < 1.0E-8. Table S2: Significant KEGG and GO categories enriched among the genes displaying alternative exon usage. Lists all KEGG pathways, GO Biological Processes and GO Molecular Function categories at FDR-adjusted P-value < 0.05.Table S3: Multi-exon genes that have significant exon-independent association with glioblastoma multiforme survival. Lists the results for the 24 multi-exon genes exhibiting expression associated with survival albeit no evidence of AEU at P-value < 5.0E-4. Table S4: Significant GO biological processes (levels 3-6) from the gene set enrichment analysis of the genome. Lists all biological processes at FDR-adjusted P-value < 0.05. Table S5: Significant GO molecular functions (levels 3-6) from the gene set enrichment analysis of the genome. Lists all molecular functions at FDR-adjusted P-value < 0.05. Table S6: Significant KEGG pathways (levels 3-6) from the gene set enrichment analysis of the genome. Lists all pathways at FDR-adjusted P-value < 0.05. Click here for file

Additional file 2 **Figure S1.** Anapc1 exon expression, moving average, and alternative splicing models. Depicts the alternative exon expression, moving average and alternative splicing models for anaphase promoting complex subunit 1. Figure S2. Herc2 exon expression, moving average, and alternative splicing models. Depicts the alternative exon expression, moving average and alternative splicing models for HECT domain and RLD domain containing E3 ubiquitin protein ligase 2. Click here for file
