# Supplementary material for: Identification and characterization of alternative exon usage linked glioblastoma multiforme survival
Source: BMC Med Genomics. 2012 Dec 4;5:59. doi: 10.1186/1755-8794-5-59 (PMC3548711; doi:10.1186/1755-8794-5-59)
Supplement: Additional file 1 — Table S1. Lists the results for the 129 multi-exon genes that exhibit evidence of AEU at P-value < 1.0E-8. Lists the results for the 129 multi-exon genes that exhibit evidence of AEU at P-value < 1.0E-8. Table S2: Significant KEGG and GO categories enriched among the genes displaying alternative exon usage. Lists all KEGG pathways, GO Biological Processes and GO Molecular Function categories at FDR-adjusted P-value < 0.05.Table S3: Multi-exon genes that have significant exon-independent association with glioblastoma multiforme survival. Lists the results for the 24 multi-exon genes exhibiting expression associated with survival albeit no evidence of AEU at P-value < 5.0E-4. Table S4: Significant GO biological processes (levels 3-6) from the gene set enrichment analysis of the genome. Lists all biological processes at FDR-adjusted P-value < 0.05. Table S5: Significant GO molecular functions (levels 3-6) from the gene set enrichment analysis of the genome. Lists all molecular functions at FDR-adjusted P-value < 0.05. Table S6: Significant KEGG pathways (levels 3-6) from the gene set enrichment analysis of the genome. Lists all pathways at FDR-adjusted P-value < 0.05. [file 1755-8794-5-59-S1.pdf]

Supplementary Table 1. Multi-exon genes that have significant (P-value < 1.0E-8) alternative exon usage associated with glioblastoma multiforme survival.

Gene Symbol	Estimate[1]	SE[2]	P-value AEU[3]	Fold Change[4]	Exon Count[5]
Ttn	0.0007	0.0001	4.2E-38	0.9993	340
Smg1	0.0017	0.0002	2.0E-24	1.0001	209
Neb	0.0007	0.0001	3.2E-21	0.9973	180
Pkd1	0.0010	0.0001	2.0E-19	1.0018	163
Herc2p2	0.0008	0.0001	2.3E-19	1.0012	163
Syne1	0.0018	0.0002	3.0E-18	0.9984	152
Snrpn	0.0018	0.0002	3.8E-18	1.0020	151
Pde4dip	0.0016	0.0002	1.3E-17	0.9993	146
Golga8c	0.0031	0.0004	4.2E-17	1.0005	141
Sspo	0.0009	0.0001	1.2E-16	1.0003	137
Ankrd36	0.0026	0.0003	1.3E-16	1.0018	137
Tbc1d3	0.0008	0.0001	2.4E-16	1.0026	135
Flj45340	0.0018	0.0002	5.5E-16	1.0007	131
Anapc1	0.0009	0.0001	5.8E-15	0.9990	122
Syne2	0.0012	0.0002	6.2E-15	1.0017	122
Nbpf10	0.0035	0.0005	1.3E-14	0.9992	118
Muc19	0.0015	0.0002	1.4E-14	1.0000	118
Obscn	0.0006	0.0001	1.5E-14	0.9999	118

Npipl3	0.0019	0.0003	4.1E-14	1.0014	114
Dst	0.0013	0.0002	9.4E-14	0.9997	111
Col7a1	0.0011	0.0001	1.4E-13	1.0001	109
Ubr4	0.0011	0.0001	1.4E-13	0.9994	109
Hmcn1	0.0006	0.0001	2.0E-13	0.9975	109
Ryr2	0.0011	0.0001	2.7E-13	0.9974	107
Macf1	0.0011	0.0002	3.1E-13	0.9975	106
Mdn1	0.0006	0.0001	3.5E-13	0.9993	106
Col4a5	0.0008	0.0001	3.5E-13	0.9992	106
Ryr1	0.0007	0.0001	4.2E-13	0.9998	105
Golga6l5	0.0013	0.0002	5.2E-13	1.0021	104
Ryr3	0.0009	0.0001	1.3E-12	0.9962	102
Dnah14	0.0007	0.0001	2.0E-12	0.9990	99
Herc2	0.0006	0.0001	3.1E-12	1.0003	97
Dnah8	0.0005	0.0001	4.7E-12	0.9997	96
Nomo1	0.0007	0.0001	4.9E-12	0.9996	95
Gpr98	0.0016	0.0002	5.9E-12	0.9948	95
Golga6a	0.0017	0.0002	7.8E-12	1.0009	93
Mycbp2	0.0009	0.0001	1.1E-11	0.9998	92
Hydin	0.0008	0.0001	1.3E-11	0.9983	92

Mll3	0.0014	0.0002	1.4E-11	0.9987	91
Kiaa1109	0.0013	0.0002	1.4E-11	0.9979	91
Dmd	0.0013	0.0002	1.7E-11	0.9996	91
Muc16	0.0010	0.0002	1.7E-11	0.9993	90
Dync2h1	0.0012	0.0002	1.9E-11	0.9984	90
Hspg2	0.0005	0.0001	2.0E-11	1.0009	90
Lrp1b	0.0006	0.0001	2.1E-11	1.0017	91
Huwe1	0.0008	0.0001	2.3E-11	1.0005	89
Vps13c	0.0013	0.0002	3.0E-11	1.0012	88
Prkdc	0.0010	0.0001	3.0E-11	0.9997	88
Lrp1	0.0008	0.0001	3.1E-11	0.9980	88
Fam21a	0.0013	0.0002	4.7E-11	1.0021	86
Golga9p	0.0019	0.0003	6.0E-11	0.9990	85
Dnah17	0.0012	0.0002	7.6E-11	0.9999	84
Dnah11	0.0006	0.0001	9.1E-11	0.9977	85
Dnah2	0.0008	0.0001	1.1E-10	0.9985	83
Dnah5	0.0008	0.0001	1.1E-10	0.9977	83
Utrn	0.0008	0.0001	1.1E-10	0.9960	83
Ddx11	0.0005	0.0001	1.2E-10	1.0014	83
Adamts7	0.0011	0.0002	1.3E-10	1.0009	82

Spdye8p	0.0011	0.0002	2.1E-10	1.0009	80
Fras1	0.0008	0.0001	2.5E-10	0.9977	80
Pkhd1l1	0.0007	0.0001	2.9E-10	1.0000	79
Lrp2	0.0005	0.0001	3.0E-10	0.9966	80
Herc1	0.0007	0.0001	3.0E-10	1.0007	79
Csmd2	0.0007	0.0001	3.1E-10	1.0028	79
Atm	0.0018	0.0003	3.5E-10	0.9999	78
Birc6	0.0010	0.0002	3.6E-10	0.9995	78
Usp34	0.0015	0.0002	3.6E-10	0.9999	78
Ush2a	0.0006	0.0001	3.6E-10	0.9997	78
Lama3	0.0013	0.0002	3.6E-10	0.9983	78
Vps13a	0.0013	0.0002	3.8E-10	0.9970	78
C12orf51	0.0006	0.0001	3.9E-10	1.0016	78
Csmd1	0.0009	0.0001	4.2E-10	1.0002	78
Dnah6	0.0007	0.0001	4.4E-10	0.9976	78
Dnah10	0.0007	0.0001	4.7E-10	0.9998	77
Dync1h1	0.0007	0.0001	4.8E-10	1.0006	77
Dnah1	0.0004	0.0001	4.9E-10	1.0007	77
Lama5	0.0004	0.0001	5.1E-10	1.0008	77
Hmcn2	0.0007	0.0001	6.1E-10	1.0005	76

Kalrn	0.0012	0.0002	6.1E-10	1.0003	76
Csmd3	0.0007	0.0001	6.2E-10	1.0038	78
Agap4	0.0007	0.0001	6.6E-10	1.0038	76
Rgpd1	0.0037	0.0006	7.4E-10	0.9997	75
Col16a1	0.0008	0.0001	7.8E-10	0.9990	75
Heatr7a	0.0006	0.0001	9.8E-10	1.0001	74
Nf1	0.0018	0.0003	9.9E-10	1.0021	74
Rnf213	0.0007	0.0001	1.0E-09	0.9979	74
Cubn	0.0006	0.0001	1.0E-09	0.9990	74
Trrap	0.0008	0.0001	1.3E-09	0.9990	73
Wdfy3	0.0009	0.0001	1.3E-09	0.9991	73
Col5a1	0.0011	0.0002	1.4E-09	0.9996	73
Dnah9	0.0008	0.0001	1.5E-09	0.9958	73
Cdh23	0.0007	0.0001	1.7E-09	1.0005	72
Pkhd1	0.0006	0.0001	1.7E-09	0.9997	72
Htt	0.0005	0.0001	1.8E-09	0.9991	72
Nbpf15	0.0019	0.0003	2.0E-09	0.9997	71
Fryl	0.0011	0.0002	2.2E-09	0.9996	71
Usp24	0.0011	0.0002	2.2E-09	0.9985	71
Fbn2	0.0009	0.0001	2.3E-09	1.0005	72

Fbn1	0.0010	0.0002	2.4E-09	0.9968	71
Cbwd1	0.0028	0.0005	2.6E-09	1.0008	70
Col11a1	0.0010	0.0002	2.6E-09	0.9997	71
Sptbn5	0.0009	0.0002	2.7E-09	1.0001	70
Stab2	0.0011	0.0002	2.7E-09	1.0002	70
Arhgap23	0.0005	0.0001	2.9E-09	0.9999	70
Nxf2	0.0012	0.0002	3.5E-09	1.0000	69
Col11a2	0.0009	0.0002	3.5E-09	1.0005	69
Dnah7	0.0006	0.0001	3.6E-09	0.9965	70
Fam90a10	0.0005	0.0001	3.7E-09	1.0007	69
Abca13	0.0010	0.0002	4.1E-09	0.9977	69
Ahnak2	0.0010	0.0002	4.2E-09	0.9955	69
Abi3bp	0.0008	0.0001	4.7E-09	0.9987	69
Reln	0.0009	0.0002	5.1E-09	0.9965	68
Myo15a	0.0014	0.0002	5.6E-09	1.0000	67
Rgpd5	0.0024	0.0004	5.7E-09	0.9996	67
Rbmy1a1	0.0010	0.0002	5.8E-09	1.0000	67
Vps13b	0.0014	0.0002	5.8E-09	0.9998	67
Wdfy4	0.0012	0.0002	5.9E-09	0.9979	67
Atrx	0.0016	0.0003	5.9E-09	0.9997	67

Col22a1	0.0011	0.0002	6.0E-09	0.9982	67
Cacna1c	0.0008	0.0001	6.2E-09	0.9997	67
Npip	0.0007	0.0001	6.3E-09	1.0018	67
Nbea	0.0009	0.0002	6.7E-09	0.9999	67
Lama1	0.0007	0.0001	6.9E-09	0.9971	67
Unc80	0.0006	0.0001	6.9E-09	0.9998	67
Dnah3	0.0008	0.0001	7.7E-09	0.9994	66
Fry	0.0011	0.0002	7.9E-09	1.0018	66
Adamts13	0.0009	0.0002	9.4E-09	1.0006	65
Tnrc18	0.0008	0.0002	9.5E-09	1.0010	65
Kiaa0467	0.0006	0.0001	9.6E-09	1.0000	65

[1]Estimate: exon-survival interaction variance indicator of alternative exon usage.

[2]SE: standard error of the estimate.

[3]P-value AEU: unadjusted P-value of alternative exon usage or exon-dependent association between expression and glioblastoma multiforme survival.

[4]Fold change: fold change in average exon expression per additional survival month.

[5]Exon Count: number of exons in the gene.

Supplementary Table 2. Significant KEGG and GO categories enriched among the genes displaying alternative exon usage.

Source	Category	Gene Count[1]	FDR P-value[2]
KEGG Pathway	(hsa04510) focal adhesion	86	3.2E-21
	(hsa04512) ecm-receptor interaction	51	8.5E-20
	(hsa02010) abc transporters	30	2.5E-12
	(hsa04810) regulation of actin cytoskeleton	66	1.7E-07
	(hsa05414) dilated cardiomyopathy	37	1.3E-06
	(hsa05412) arrhythmogenic right ventricular cardiomyopathy (ARVC)	32	5.9E-06
	(hsa04070) phosphatidylinositol signaling system	31	1.2E-05
	(hsa05410) hypertrophic cardiomyopathy (HCM)	32	1.3E-04
	(hsa05222) small cell lung cancer	31	3.6E-04
GO Biological Process	(GO:0051056) regulation of small GTPase mediated signal transduction	105	5.0E-25
	(GO:0007155) cell adhesion	197	2.3E-22
	(GO:0022610) biological adhesion	197	2.7E-22
	(GO:0007010) cytoskeleton organization	129	1.3E-15
	(GO:0035023) regulation of Rho protein signal transduction	51	1.7E-15
	(GO:0046578) regulation of Ras protein signal transduction	79	5.0E-15
	(GO:0030029) actin filament-	85	2.3E-14

based process		
(GO:0030030) cell projection organization	109	1.2E-12
(GO:0051276) chromosome organization	132	1.4E-12
(GO:0016568) chromatin modification	89	1.9E-12
(GO:0007018) microtubule-based movement	51	2.1E-12
(GO:0007017) microtubule-based process	82	3.0E-11
(GO:0030036) actin cytoskeleton organization	76	3.4E-11
(GO:0007229) integrin-mediated signaling pathway	36	4.5E-10
(GO:0000902) cell morphogenesis	100	8.0E-10
(GO:0006468) protein amino acid phosphorylation	156	3.3E-09
(GO:0032989) cellular component morphogenesis	106	4.8E-09
(GO:0031589) cell-substrate adhesion	42	7.2E-09
(GO:0000059) protein import into nucleus, docking	16	1.7E-08
(GO:0016192) vesicle-mediated transport	137	2.5E-08
(GO:0007160) cell-matrix adhesion	38	1.0E-07
(GO:0050657) nucleic acid transport	40	1.0E-07
(GO:0050658) RNA transport	40	1.0E-07
(GO:0051236) establishment of RNA localization	40	1.0E-07

(GO:0043062) extracellular structure organization	55	1.7E-07
(GO:0048858) cell projection morphogenesis	72	2.3E-07
(GO:0006403) RNA localization	40	3.1E-07
(GO:0046907) intracellular transport	147	4.7E-07
(GO:0006325) chromatin organization	97	4.8E-07
(GO:0032990) cell part morphogenesis	73	7.5E-07
(GO:0000904) cell morphogenesis involved in differentiation	70	1.4E-06
(GO:0006793) phosphorus metabolic process	197	2.8E-06
(GO:0006796) phosphate metabolic process	197	2.8E-06
(GO:0048666) neuron development	87	4.4E-06
(GO:0031175) neuron projection development	71	4.9E-06
(GO:0048812) neuron projection morphogenesis	62	7.6E-06
(GO:0048667) cell morphogenesis involved in neuron differentiation	61	9.3E-06
(GO:0008104) protein localization	179	1.5E-05
(GO:0051028) mRNA transport	34	1.7E-05
(GO:0015931) nucleobase, nucleoside, nucleotide and nucleic acid transport	40	1.9E-05
(GO:0030705) cytoskeleton-	25	2.0E-05

	dependent intracellular transport		
	(GO:0016310) phosphorylation	164	3.4E-05
	(GO:0050954) sensory perception of mechanical stimulus	37	4.8E-05
	(GO:0007242) intracellular signaling cascade	236	5.9E-05
	(GO:0006816) calcium ion transport	45	9.0E-05
	(GO:0007605) sensory perception of sound	35	1.1E-04
	(GO:0006913) nucleocytoplasmic transport	47	2.4E-04
	(GO:0043087) regulation of GTPase activity	40	2.6E-04
	(GO:0022403) cell cycle phase	95	2.9E-04
	(GO:0007409) axonogenesis	54	3.6E-04
	(GO:0051169) nuclear transport	47	3.6E-04
	(GO:0015914) phospholipid transport	18	3.8E-04
	(GO:0000279) M phase	79	5.3E-04
	(GO:0006338) chromatin remodeling	24	5.3E-04
	(GO:0030182) neuron differentiation	98	6.3E-04
	(GO:0033043) regulation of organelle organization	58	6.4E-04
	(GO:0016044) membrane organization	88	6.4E-04
	(GO:0030198) extracellular matrix organization	35	7.2E-04
	(GO:0046034) ATP metabolic process	35	9.3E-04
**GO**	(GO:0032559) adenyl	437	2.0E-59

**Molecular Function**	ribonucleotide binding		
	(GO:0005524) ATP binding	433	2.2E-59
	(GO:0030554) adenyl nucleotide binding	451	9.9E-59
	(GO:0001882) nucleoside binding	456	6.3E-58
	(GO:0001883) purine nucleoside binding	451	1.5E-56
	(GO:0032555) purine ribonucleotide binding	466	2.9E-44
	(GO:0032553) ribonucleotide binding	466	2.9E-44
	(GO:0017076) purine nucleotide binding	480	5.2E-44
	(GO:0000166) nucleotide binding	523	7.4E-39
	(GO:0003774) motor activity	86	1.3E-34
	(GO:0016887) ATPase activity	139	3.3E-33
	(GO:0030695) GTPase regulator activity	155	2.1E-32
	(GO:0060589) nucleoside-triphosphatase regulator activity	156	1.0E-31
	(GO:0008092) cytoskeletal protein binding	168	8.0E-27
	(GO:0003779) actin binding	125	1.7E-25
	(GO:0005083) small GTPase regulator activity	112	2.4E-25
	(GO:0042623) ATPase activity, coupled	108	4.2E-23
	(GO:0005085) guanyl-nucleotide exchange factor activity	72	8.4E-20
	(GO:0003777) microtubule motor activity	46	7.9E-17

(GO:0005089) Rho guanyl-nucleotide exchange factor activity	44	7.2E-16
(GO:0005516) calmodulin binding	62	6.6E-15
(GO:0005088) Ras guanyl-nucleotide exchange factor activity	47	1.3E-14
(GO:0004386) helicase activity	61	3.3E-14
(GO:0005509) calcium ion binding	217	7.7E-14
(GO:0005096) GTPase activator activity	78	1.4E-12
(GO:0051020) GTPase binding	50	3.4E-12
(GO:0042626) ATPase activity, coupled to transmembrane movement of substances	49	1.8E-11
(GO:0043492) ATPase activity, coupled to movement of substances	49	2.7E-11
(GO:0016820) hydrolase activity, acting on acid anhydrides, catalyzing transmembrane movement of substances	49	4.2E-11
(GO:0005201) extracellular matrix structural constituent	41	3.2E-10
(GO:0031267) small GTPase binding	45	3.6E-10
(GO:0004672) protein kinase activity	149	6.9E-10
(GO:0015399) primary active transmembrane transporter activity	49	3.0E-09
(GO:0015405) P-P-bond-hydrolysis-driven transmembrane transporter	49	3.0E-09

activity		
(GO:0017016) Ras GTPase binding	41	3.2E-09
(GO:0019899) enzyme binding	130	1.2E-08
(GO:0004012) phospholipid-translocating ATPase activity	15	1.8E-08
(GO:0015247) aminophospholipid transporter activity	15	1.8E-08
(GO:0000146) microfilament motor activity	16	1.9E-08
(GO:0019992) diacylglycerol binding	33	5.6E-08
(GO:0008047) enzyme activator activity	92	7.4E-08
(GO:0015662) ATPase activity, coupled to transmembrane movement of ions, phosphorylative mechanism	28	1.9E-07
(GO:0005548) phospholipid transporter activity	20	3.2E-07
(GO:0005262) calcium channel activity	34	1.4E-06
(GO:0017048) Rho GTPase binding	22	3.3E-06
(GO:0070035) purine NTP-dependent helicase activity	37	1.5E-05
(GO:0008026) ATP-dependent helicase activity	37	1.5E-05
(GO:0004674) protein serine/threonine kinase activity	103	1.8E-05
(GO:0051015) actin filament binding	25	4.1E-05
(GO:0019198) transmembrane receptor protein phosphatase activity	14	4.3E-05

(GO:0005099) Ras GTPase activator activity	34	9.1E-05
(GO:0000287) magnesium ion binding	104	1.4E-04
(GO:0046872) metal ion binding	668	1.5E-04
(GO:0008307) structural constituent of muscle	21	2.2E-04
(GO:0005198) structural molecule activity	134	3.5E-04
(GO:0043169) cation binding	670	3.8E-04
(GO:0004714) transmembrane receptor protein tyrosine kinase activity	27	4.9E-04
(GO:0043167) ion binding	678	5.1E-04

[1]Gene Count: number of genes that have significant alternative exon usage within category.

[2]FDR P-value: False discovery rate adjusted P-value of the hypergeometric test of category enrichment.

Supplementary Table 3. Multi-exon genes that have significant exon-independent association with glioblastoma multiforme survival.

Gene Symbol	Estimate[1]	SE[2]	Fold Change[3]	P-value[4]	Exon Count[5]
Igkc	-0.0455	0.0072	0.9690	1.4E-09	44
C7orf28a	-0.0501	0.0098	0.9659	7.2E-07	33
Pcdh15	0.0249	0.0050	1.0174	1.4E-06	46
Flnc	-0.0123	0.0027	0.9915	8.2E-06	48
Cdc42	-0.0540	0.0124	0.9633	2.1E-05	25
Gnas	-0.0598	0.0139	0.9594	2.6E-05	30
Znf32	0.0063	0.0015	1.0044	4.8E-05	02
Prr20a	-0.0301	0.0075	0.9794	7.5E-05	25
Sema3e	-0.0256	0.0066	0.9824	1.3E-04	18
Golga8j	-0.0536	0.0141	0.9635	1.8E-04	20
Sptlc1	-0.0424	0.0112	0.9710	2.0E-04	29
Sptbn1	-0.0234	0.0063	0.9839	2.6E-04	46
Xage1a	-0.0368	0.0099	0.9748	2.6E-04	25
Tbc1d5	-0.0374	0.0102	0.9744	3.1E-04	31
Sirt2	0.0337	0.0092	1.0236	3.2E-04	17
C7orf58	-0.0279	0.0076	0.9809	3.3E-04	29
Six1	0.0056	0.0015	1.0039	3.3E-04	05
Rpsa	-0.0535	0.0148	0.9636	3.7E-04	23
Loc100289340	0.0064	0.0018	1.0045	3.7E-04	02
LOC100289627	0.0079	0.0022	1.0055	3.8E-04	02
Snord116-3	0.0087	0.0024	1.0060	3.8E-04	02

Dync1i2	-0.0326	0.0091	0.9777	4.2E-04	27
Zdhhc20	-0.0392	0.0110	0.9732	4.6E-04	26
Appl1	-0.0446	0.0126	0.9695	4.9E-04	28

[1]Estimate: change in average exon expression per additional survival month (in log2 units).

[2]SE: standard error of the estimate.

[3]Fold change: fold change in average exon expression per additional survival month.

[4]P-value: unadjusted P-value of the change in average exon expression per additional survival month.

[5]Exon Count: number of exons in the gene.

Supplementary Table 4. Significant GO biological processes (levels 3-6) from the gene set enrichment analysis of the genome.

GO Identifier	GO Biological Process	Over-Expressed Gene[1]	Under-Expressed Genes[2]	Log Odds Ratio[3]	FDR P-value[4]
GO:0046907	intracellular transport	357	560	-0.7338	3.8E-24
GO:0034613	cellular protein localization	245	433	-0.84s90	4.8E-24
GO:0043067	regulation of programmed cell death	351	490	-0.6110	1.7E-15
GO:0016192	vesicle-mediated transport	271	400	-0.6639	1.2E-14
GO:0006629	lipid metabolic process	424	538	-0.5148	1.3E-12
GO:0044265	cellular macromolecule catabolic process	373	485	-0.5379	2.1E-12
GO:0044255	cellular lipid metabolic process	346	457	-0.5528	2.4E-12
GO:0050793	regulation of developmental process	442	549	-0.4932	4.3E-12
GO:0007049	cell cycle	418	522	-0.4978	1.1E-11
GO:0009966	regulation of signal transduction	414	509	-0.4812	9.9E-11
GO:0051169	nuclear transport	128	213	-0.7766	2.2E-10
GO:0005975	carbohydrate metabolic process	294	381	-0.5304	1.1E-09
GO:0006928	cellular component movement	306	391	-0.5164	1.6E-09
GO:0010942	positive regulation of cell death	176	259	-0.6545	1.6E-09

GO:0006461	protein complex assembly	303	384	-0.5077	4.0E-09
GO:0050790	regulation of catalytic activity	403	480	-0.4475	4.5E-09
GO:0016044	cellular membrane organization	183	261	-0.6229	6.0E-09
GO:0051246	regulation of protein metabolic process	236	313	-0.5513	1.2E-08
GO:0009893	positive regulation of metabolic process	402	474	-0.4369	1.3E-08
GO:0051716	cellular response to stimulus	406	475	-0.4289	2.1E-08
GO:0019752	carboxylic acid metabolic process	306	379	-0.4841	2.2E-08
GO:0006082	organic acid metabolic process	308	381	-0.4829	2.3E-08
GO:0033554	cellular response to stress	284	357	-0.4984	2.5E-08
GO:0006091	generation of precursor metabolites and energy	175	247	-0.6117	2.7E-08
GO:0010033	response to organic substance	361	430	-0.4459	3.3E-08
GO:0033365	protein localization in organelle	76	136	-0.8458	7.9E-08
GO:0043085	positive regulation of catalytic activity	241	309	-0.5168	1.0E-07
GO:0016477	cell migration	190	255	-0.5611	2.1E-07
GO:0000278	mitotic cell cycle	212	276	-0.5310	2.5E-07
GO:0006066	alcohol metabolic process	241	304	-0.5000	2.8E-07

GO:0006519	cellular amino acid and derivative metabolic process	196	260	-0.5494	2.8E-07
GO:0022402	cell cycle process	290	352	-0.4625	2.8E-07
GO:0048193	Golgi vesicle transport	38	86	-1.0788	2.8E-07
GO:0008610	lipid biosynthetic process	175	237	-0.5695	3.7E-07
GO:0009308	amine metabolic process	224	285	-0.5080	5.2E-07
GO:0006575	cellular amino acid derivative metabolic process	77	131	-0.7948	5.6E-07
GO:0002682	regulation of immune system process	183	241	-0.5413	9.8E-07
GO:0055114	oxidation reduction	321	375	-0.4242	1.1E-06
GO:0051128	regulation of cellular component organization	214	272	-0.5065	1.1E-06
GO:0048870	cell motility	231	287	-0.4839	1.4E-06
GO:0007600	sensory perception	546	288	0.3954	1.5E-06
GO:0051726	regulation of cell cycle	144	199	-0.5884	2.1E-06
GO:0044262	cellular carbohydrate metabolic process	180	235	-0.5323	2.4E-06
GO:0008361	regulation of cell size	114	167	-0.6459	2.5E-06
GO:0015980	energy derivation by oxidation of organic compounds	85	135	-0.7258	2.6E-06
GO:0048468	cell development	455	493	-0.3504	2.7E-06

GO:0000902	cell morphogenesis	209	262	-0.4921	2.8E-06
GO:0040008	regulation of growth	161	215	-0.5544	2.8E-06
GO:0006644	phospholipid metabolic process	99	150	-0.6791	3.1E-06
GO:0002684	positive regulation of immune system process	112	161	-0.6266	7.8E-06
GO:0006897	endocytosis	122	171	-0.6015	7.8E-06
GO:0022900	electron transport chain	55	97	-0.8292	1.2E-05
GO:0055085	transmembrane transport	92	138	-0.6684	1.2E-05
GO:0006955	immune response	431	462	-0.3384	1.3E-05
GO:0043623	cellular protein complex assembly	110	156	-0.6127	1.4E-05
GO:0009892	negative regulation of metabolic process	350	387	-0.3682	1.6E-05
GO:0045926	negative regulation of growth	45	84	-0.8855	1.9E-05
GO:0030030	cell projection organization	221	265	-0.4471	2.0E-05
GO:0006954	inflammatory response	222	266	-0.4464	2.0E-05
GO:0045333	cellular respiration	50	90	-0.8493	2.0E-05
GO:0009628	response to abiotic stimulus	303	342	-0.3879	2.1E-05
GO:0044419	interspecies interaction between organisms	157	202	-0.5163	2.1E-05
GO:0022008	neurogenesis	304	343	-0.3876	2.1E-05

GO:0042127	regulation of cell proliferation	402	431	-0.3378	2.9E-05
GO:0045595	regulation of cell differentiation	244	285	-0.4211	3.0E-05
GO:0008284	positive regulation of cell proliferation	212	252	-0.4378	5.1E-05
GO:0019725	cellular homeostasis	256	292	-0.3971	6.8E-05
GO:0051301	cell division	135	176	-0.5286	7.2E-05
GO:0005996	monosaccharide metabolic process	113	153	-0.5659	7.4E-05
GO:0007010	cytoskeleton organization	257	292	-0.3932	8.1E-05
GO:0006812	cation transport	385	409	-0.3277	8.8E-05
GO:0006952	defense response	416	436	-0.3145	9.2E-05
GO:0006974	response to DNA damage stimulus	201	238	-0.4334	1.0E-04
GO:0009790	embryonic development	292	323	-0.3668	1.1E-04
GO:0016311	dephosphorylation	92	130	-0.6080	1.1E-04
GO:0009611	response to wounding	374	397	-0.3266	1.2E-04
GO:0051270	regulation of cellular component movement	96	134	-0.5958	1.2E-04
GO:0051186	cofactor metabolic process	123	161	-0.5321	1.3E-04
GO:0002443	leukocyte mediated immunity	102	139	-0.5719	1.4E-04
GO:0002237	response to molecule of bacterial origin	42	74	-0.8270	1.6E-04

GO:0045597	positive regulation of cell differentiation	112	149	-0.5480	1.7E-04
GO:0009890	negative regulation of biosynthetic process	285	314	-0.3624	1.7E-04
GO:0051347	positive regulation of transferase activity	113	150	-0.5458	1.7E-04
GO:0051351	positive regulation of ligase activity	34	65	-0.9084	1.8E-04
GO:0034984		186	220	-0.4317	2.0E-04
GO:0002250	adaptive immune response	94	130	-0.5863	2.0E-04
GO:0050900	leukocyte migration	35	65	-0.8793	2.0E-04
GO:0030334	regulation of cell migration	86	121	-0.6033	2.4E-04
GO:0046486	glycerolipid metabolic process	81	115	-0.6121	2.7E-04
GO:0051438	regulation of ubiquitin-protein ligase activity	37	67	-0.8541	2.7E-04
GO:0016050	vesicle organization	33	62	-0.8908	2.9E-04
GO:0050890	cognition	590	346	0.2872	2.9E-04
GO:0051789	response to protein stimulus	55	87	-0.7195	2.9E-04
GO:0002520	immune system development	188	220	-0.4209	3.0E-04
GO:0009887	organ morphogenesis	398	412	-0.3012	3.0E-04
GO:0009792	embryonic development ending in birth or egg hatching	175	207	-0.4314	3.2E-04

GO:0007005	mitochondrion organization	96	65	-0.7394	3.2E-04
GO:0048878	chemical homeostasis	286	310	-0.3457	3.5E-04
GO:0006979	response to oxidative stress	127	79	-0.6552	3.6E-04
GO:0034101	erythrocyte homeostasis	33	61	-0.8744	3.9E-04
GO:0050867	positive regulation of cell activation	58	89	-0.6891	3.9E-04
GO:0006403	RNA localization	48	78	-0.7461	4.2E-04
GO:0051272	positive regulation of cellular component movement	48	78	-0.7461	4.2E-04
GO:0051402	neuron apoptosis	51	81	-0.7233	4.4E-04
GO:0051325	interphase	60	91	-0.6774	4.6E-04
GO:0019318	hexose metabolic process	144	86	-0.6148	4.8E-04
GO:0043009	chordate embryonic development	174	204	-0.4223	5.1E-04
GO:0050777	negative regulation of immune response	13	35	-1.2495	5.1E-04
GO:0009607	response to biotic stimulus	207	235	-0.3906	5.2E-04
GO:0030036	actin cytoskeleton organization	146	177	-0.4553	5.3E-04
GO:0007568	aging	59	89	-0.6719	5.3E-04
GO:0007610	behavior	282	303	-0.3366	5.4E-04
GO:0031667	response to nutrient levels	110	141	-0.5103	6.0E-04
GO:0040012	regulation of locomotion	102	133	-0.5272	6.0E-04

GO:0030029	actin filament-based process	155	185	-0.4398	6.1E-04
GO:0055082	cellular chemical homeostasis	217	242	-0.3727	6.8E-04
GO:0050776	regulation of immune response	114	144	-0.4956	7.4E-04
GO:0009268	response to pH	8	27	-1.4751	8.4E-04
GO:0045321	leukocyte activation	184	210	-0.3953	9.8E-04
GO:0048872	homeostasis of number of cells	63	91	-0.6284	9.8E-04
GO:0006259	DNA metabolic process	323	336	-0.3043	1.1E-03
GO:0014075	response to amine stimulus	23	46	-0.9526	1.1E-03
GO:0043434	response to peptide hormone stimulus	89	118	-0.5434	1.1E-03
GO:0006084	acetyl-CoA metabolic process	16	37	-1.0974	1.1E-03
GO:0006800	oxygen and reactive oxygen species metabolic process	39	65	-0.7709	1.1E-03
GO:0050801	ion homeostasis	237	258	-0.3487	1.1E-03
GO:0001775	cell activation	208	231	-0.3682	1.2E-03
GO:0009411	response to UV	28	52	-0.8787	1.2E-03
GO:0002526	acute inflammatory response	88	116	-0.5375	1.2E-03
GO:0009991	response to extracellular stimulus	123	150	-0.4603	1.3E-03
GO:0046903	secretion	241	260	-0.3395	1.4E-03
GO:0046483	heterocycle metabolic process	245	263	-0.3345	1.6E-03
GO:0060348	bone development	100	127	-0.5004	1.6E-03

GO:0001501	skeletal system development	198	220	-0.3684	1.6E-03
GO:0007566	embryo implantation	16	36	-1.0699	1.6E-03
GO:0008015	blood circulation	128	154	-0.4468	1.6E-03
GO:0008202	steroid metabolic process	119	145	-0.4593	1.6E-03
GO:0009314	response to radiation	101	128	-0.4983	1.6E-03
GO:0009408	response to heat	30	53	-0.8287	1.6E-03
GO:0042113	B cell activation	96	61	-0.6752	1.7E-03
GO:0042274	ribosomal small subunit biogenesis	2	15	-2.2730	1.8E-03
GO:0006869	lipid transport	83	109	-0.5335	1.8E-03
GO:0050778	positive regulation of immune response	76	102	-0.5550	1.8E-03
GO:0010243	response to organic nitrogen	39	63	-0.7394	1.9E-03
GO:0006749	glutathione metabolic process	14	33	-1.1163	2.2E-03
GO:0030031	cell projection assembly	58	83	-0.6187	2.2E-03
GO:0033993	response to lipid	11	29	-1.2281	2.2E-03
GO:0009266	response to temperature stimulus	169	191	-0.3848	2.2E-03
GO:0042594	response to starvation	24	45	-0.8879	2.4E-03
GO:0051707	response to other organism	156	178	-0.3940	2.5E-03
GO:0043087	regulation of GTPase activity	67	92	-0.5776	2.6E-03
GO:0008643	carbohydrate transport	48	72	-0.6655	2.7E-03
GO:0006766	vitamin metabolic	58	82	-0.6065	2.8E-03

	process				
GO:0009725	response to hormone stimulus	202	220	-0.3481	2.8E-03
GO:0034097	response to cytokine stimulus	43	66	-0.6883	2.9E-03
GO:0000910	cytokinesis	24	44	-0.8653	3.4E-03
GO:0001816	cytokine production	110	133	-0.4511	3.5E-03
GO:0007611	learning or memory	61	84	-0.5802	3.5E-03
GO:0051099	positive regulation of binding	42	64	-0.6809	3.5E-03
GO:0010035	response to inorganic substance	84	107	-0.5027	3.5E-03
GO:0005976	polysaccharide metabolic process	81	104	-0.5106	3.7E-03
GO:0002367	cytokine production involved in immune response	13	30	-1.0949	3.7E-03
GO:0006081	cellular aldehyde metabolic process	13	30	-1.0949	3.7E-03
GO:0042692	muscle cell differentiation	59	82	-0.5894	3.7E-03
GO:0070201	regulation of establishment of protein localization	66	89	-0.5593	3.7E-03
GO:0002467	germinal center formation	4	17	-1.7050	3.9E-03
GO:0019682	glyceraldehyde-3-phosphate metabolic process	0	6	1.79E308	4.0E-03
GO:0006937	regulation of muscle contraction	38	59	-0.6995	4.0E-03
GO:0006790	sulfur metabolic process	65	87	-0.5517	4.4E-03

GO:0032943	mononuclear cell proliferation	65	87	-0.5517	4.4E-03
GO:0043086	negative regulation of catalytic activity	202	103	-0.4564	4.8E-03
GO:0001819	positive regulation of cytokine production	43	64	-0.6573	4.9E-03
GO:0042254	ribosome biogenesis	67	89	-0.5442	5.0E-03
GO:0007155	cell adhesion	500	477	-0.2183	5.1E-03
GO:0046651	lymphocyte proliferation	64	86	-0.5556	5.2E-03
GO:0001817	regulation of cytokine production	94	115	-0.4624	5.5E-03
GO:0048002	antigen processing and presentation of peptide antigen	15	32	-1.0164	5.6E-03
GO:0051271	negative regulation of cellular component movement	36	56	-0.7013	5.7E-03
GO:0002504	antigen processing and presentation of peptide or polysaccharide antigen via MHC	10	25	-1.1747	5.8E-03
GO:0007160	cell-matrix adhesion	74	95	-0.5101	5.8E-03
GO:0007507	heart development	120	140	-0.4153	5.8E-03
GO:0031669	cellular response to nutrient levels	26	45	-0.8077	5.9E-03
GO:0009888	tissue development	414	402	-0.2351	5.9E-03
GO:0009100	glycoprotein metabolic process	112	132	-0.4253	6.2E-03
GO:0010212	response to ionizing radiation	29	48	-0.7631	6.2E-03
GO:0043542	endothelial cell	24	42	-0.8186	6.2E-03

	migration				
GO:0015669	gas transport	9	24	-1.2392	6.3E-03
GO:0046164	alcohol catabolic process	57	78	-0.5736	6.4E-03
GO:0050880	regulation of blood vessel size	50	70	-0.5962	6.4E-03
GO:0051591	response to cAMP	27	46	-0.7919	6.6E-03
GO:0006839	mitochondrial transport	64	43	-0.7298	6.6E-03
GO:0043388	positive regulation of DNA binding	38	58	-0.6823	6.7E-03
GO:0032940	secretion by cell	200	212	-0.3204	6.9E-03
GO:0060047	heart contraction	52	72	-0.5852	7.3E-03
GO:0051604	protein maturation	67	87	-0.5213	7.4E-03
GO:0007033	vacuole organization	15	31	-0.9846	7.8E-03
GO:0045787	positive regulation of cell cycle	34	53	-0.7032	7.9E-03
GO:0000279	M phase	193	205	-0.3223	7.9E-03
GO:0042743	hydrogen peroxide metabolic process	17	33	-0.9220	7.9E-03
GO:0001944	vasculature development	161	176	-0.3507	8.0E-03
GO:0032880	regulation of protein localization	77	97	-0.4912	8.0E-03
GO:0048871	multicellular organismal homeostasis	177	190	-0.3326	8.6E-03
GO:0042493	response to drug	140	156	-0.3694	8.6E-03
GO:0006900	membrane budding	12	27	-1.0694	8.7E-03
GO:0035265	organ growth	27	45	-0.7699	8.7E-03
GO:0048660	regulation of smooth muscle cell	30	48	-0.7291	9.0E-03

	proliferation				
GO:0007346	regulation of mitotic cell cycle	80	99	-0.4733	9.0E-03
GO:0015851	nucleobase transport	2	12	-2.0496	9.0E-03
GO:0007584	response to nutrient	85	104	-0.4621	9.2E-03
GO:0046649	lymphocyte activation	162	176	-0.3444	9.3E-03
GO:0016042	lipid catabolic process	111	128	-0.4032	9.7E-03
GO:0006936	muscle contraction	112	129	-0.4020	9.8E-03
GO:0031122	cytoplasmic microtubule organization	3	14	-1.7984	9.8E-03
GO:0060263	regulation of respiratory burst	3	14	-1.7984	9.8E-03
GO:0070542	response to fatty acid	3	14	-1.7984	9.8E-03
GO:0042110	T cell activation	113	130	-0.4009	1.0E-02
GO:0009617	response to bacterium	103	120	-0.4133	1.0E-02
GO:0006112	energy reserve metabolic process	41	59	-0.6233	1.1E-02
GO:0006516	glycoprotein catabolic process	13	28	-1.0257	1.1E-02
GO:0010038	response to metal ion	75	93	-0.4752	1.1E-02
GO:0031668	cellular response to extracellular stimulus	38	56	-0.6470	1.1E-02
GO:0048659	smooth muscle cell proliferation	32	49	-0.6851	1.1E-02
GO:0034976	response to endoplasmic reticulum stress	22	38	-0.8053	1.1E-02

GO:0046677	response to antibiotic	30	47	-0.7080	1.2E-02
GO:0016197	endosome transport	33	50	-0.6746	1.2E-02
GO:0009615	response to virus	65	83	-0.5043	1.2E-02
GO:0002507	tolerance induction	7	19	-1.2566	1.2E-02
GO:0031529	ruffle organization	7	19	-1.2566	1.2E-02
GO:0042157	lipoprotein metabolic process	50	68	-0.5670	1.2E-02
GO:0006941	striated muscle contraction	40	57	-0.6134	1.2E-02
GO:0009116	nucleoside metabolic process	40	57	-0.6134	1.2E-02
GO:0045444	fat cell differentiation	37	54	-0.6372	1.2E-02
GO:0001659	temperature homeostasis	140	153	-0.3498	1.3E-02
GO:0002474	antigen processing and presentation of peptide antigen via MHC class I	9	22	-1.1520	1.3E-02
GO:0051341	regulation of oxidoreductase activity	26	42	-0.7384	1.3E-02
GO:0051051	negative regulation of transport	77	94	-0.4595	1.3E-02
GO:0042891	antibiotic transport	19	34	-0.8405	1.4E-02
GO:0006986	response to unfolded protein	42	59	-0.5991	1.4E-02
GO:0019884	antigen processing and presentation of exogenous antigen	11	24	-1.0384	1.4E-02
GO:0042088	T-helper 1 type immune response	11	24	-1.0384	1.4E-02
GO:0051409	response to nitrosative stress	1	9	-2.4548	1.4E-02

GO:0032026	response to magnesium ion	2	11	-1.9625	1.5E-02
GO:0048568	embryonic organ development	33	49	-0.6543	1.5E-02
GO:0051608	histamine transport	2	11	-1.9625	1.5E-02
GO:0032637	interleukin-8 production	8	20	-1.1744	1.5E-02
GO:0006643	membrane lipid metabolic process	47	64	-0.5681	1.5E-02
GO:0009416	response to light stimulus	76	92	-0.4509	1.5E-02
GO:0033194	response to hydroperoxide	3	13	-1.7242	1.5E-02
GO:0001666	response to hypoxia	86	102	-0.4307	1.6E-02
GO:0031589	cell-substrate adhesion	92	107	-0.4112	1.6E-02
GO:0006518	peptide metabolic process	56	72	-0.5108	1.6E-02
GO:0007032	endosome organization	10	23	-1.0912	1.6E-02
GO:0042089	cytokine biosynthetic process	52	68	-0.5277	1.6E-02
GO:0042092	T-helper 2 type immune response	10	23	-1.0912	1.6E-02
GO:0048565	digestive tract development	23	38	-0.7608	1.6E-02
GO:0006939	smooth muscle contraction	38	54	-0.6105	1.6E-02
GO:0042158	lipoprotein biosynthetic process	38	54	-0.6105	1.6E-02
GO:0048646	anatomical structure formation involved in morphogenesis	211	215	-0.2804	1.6E-02

GO:0007267	cell-cell signaling	391	371	-0.2108	1.7E-02
GO:0002437	inflammatory response to antigenic stimulus	42	58	-0.5820	1.7E-02
GO:0008038	neuron recognition	14	27	-0.9151	1.7E-02
GO:0033014	tetrapyrrole biosynthetic process	14	27	-0.9151	1.7E-02
GO:0007517	muscle organ development	164	173	-0.3145	1.8E-02
GO:0035239	tube morphogenesis	76	91	-0.4399	1.8E-02
GO:0016568	chromatin modification	151	161	-0.3250	1.8E-02
GO:0046148	pigment biosynthetic process	27	42	-0.7006	1.8E-02
GO:0007623	circadian rhythm	33	48	-0.6336	1.8E-02
GO:0045176	apical protein localization	9	21	-1.1054	1.8E-02
GO:0046688	response to copper ion	16	16	-1.1256	1.9E-02
GO:0001967	suckling behavior	6	17	-1.2994	1.9E-02
GO:0008105	asymmetric protein localization	11	23	-0.9958	2.0E-02
GO:0014823	response to activity	11	23	-0.9958	2.0E-02
GO:0009791	post-embryonic development	41	56	-0.5708	2.0E-02
GO:0060021	palate development	20	34	-0.7892	2.0E-02
GO:0030198	extracellular matrix organization	57	72	-0.4930	2.1E-02
GO:0050673	epithelial cell proliferation	45	60	-0.5468	2.1E-02

GO:0050678	regulation of epithelial cell proliferation	38	53	-0.5917	2.1E-02
GO:0042107	cytokine metabolic process	53	68	-0.5085	2.1E-02
GO:0032675	regulation of interleukin-6 production	23	37	-0.7340	2.1E-02
GO:0050665	hydrogen peroxide biosynthetic process	5	15	-1.3565	2.1E-02
GO:0051642	centrosome localization	5	15	-1.3565	2.1E-02
GO:0006929	substrate-bound cell migration	8	19	-1.1230	2.1E-02
GO:0042119	neutrophil activation	8	19	-1.1230	2.1E-02
GO:0048278	vesicle docking	18	31	-0.8020	2.1E-02
GO:0031344	regulation of cell projection organization	50	65	-0.5216	2.1E-02
GO:0051345	positive regulation of hydrolase activity	98	111	-0.3846	2.2E-02
GO:0048732	gland development	78	92	-0.4248	2.3E-02
GO:0007031	peroxisome organization	12	24	-0.9513	2.3E-02
GO:0007162	negative regulation of cell adhesion	24	38	-0.7182	2.3E-02
GO:0007417	central nervous system development	240	238	-0.2533	2.3E-02
GO:0008285	negative regulation of cell proliferation	197	200	-0.2764	2.3E-02
GO:0016048	detection of temperature stimulus	3	12	-1.6440	2.3E-02

GO:0043206	fibril organization	3	12	-1.6440	2.3E-02
GO:0045175	basal protein localization	0	6	1.79E308	2.3E-02
GO:0045216	cell-cell junction organization	19	32	-0.7797	2.3E-02
GO:0006999	nuclear pore organization	2	10	-1.8671	2.4E-02
GO:0009414	response to water deprivation	2	10	-1.8671	2.4E-02
GO:0043254	regulation of protein complex assembly	68	41	-0.6210	2.4E-02
GO:0015893	drug transport	22	35	-0.7228	2.4E-02
GO:0019886	antigen processing and presentation of exogenous peptide antigen via MHC class I	7	18	-1.2025	2.4E-02
GO:0042116	macrophage activation	17	30	-0.8264	2.4E-02
GO:0055093	response to hyperoxia	7	18	-1.2025	2.4E-02
GO:0051046	regulation of secretion	116	127	-0.3509	2.5E-02
GO:0034405	response to fluid shear stress	9	20	-1.0565	2.5E-02
GO:0070555	response to interleukin-1	9	20	-1.0565	2.5E-02
GO:0006944	cellular membrane fusion	35	49	-0.5953	2.6E-02
GO:0015914	phospholipid transport	20	33	-0.7592	2.6E-02
GO:0009101	glycoprotein biosynthetic process	94	106	-0.3800	2.6E-02
GO:0009595	detection of biotic stimulus	13	25	-0.9121	2.6E-02

GO:0045454	cell redox homeostasis	39	53	-0.5657	2.6E-02
GO:0046620	regulation of organ growth	23	36	-0.7065	2.6E-02
GO:0048771	tissue remodeling	43	57	-0.5409	2.7E-02
GO:0032092	positive regulation of protein binding	6	16	-1.2387	2.7E-02
GO:0046209	nitric oxide metabolic process	26	39	-0.6640	2.7E-02
GO:0007017	microtubule-based process	149	156	-0.3065	2.8E-02
GO:0045471	response to ethanol	40	54	-0.5591	2.8E-02
GO:0001776	leukocyte homeostasis	37	26	-0.7733	2.8E-02
GO:0014070	response to organic cyclic substance	81	93	-0.3978	2.9E-02
GO:0006935	chemotaxis	123	132	-0.3308	2.9E-02
GO:0050826	response to freezing	130	138	-0.3200	2.9E-02
GO:0035272	exocrine system development	14	26	-0.8773	3.0E-02
GO:0046685	response to arsenic	8	18	-1.0689	3.0E-02
GO:0007617	mating behavior	12	23	-0.9087	3.1E-02
GO:0035264	multicellular organism growth	54	67	-0.4749	3.1E-02
GO:0043330	response to exogenous dsRNA	10	21	-1.0000	3.1E-02
GO:0051668	localization within membrane	10	21	-1.0000	3.1E-02
GO:0050905	neuromuscular process	38	51	-0.5531	3.1E-02
GO:0006959	humoral immune response	50	63	-0.4902	3.1E-02
GO:0031346	positive regulation of cell projection	28	41	-0.6400	3.1E-02

	organization				
GO:0001889	liver development	35	48	-0.5746	3.2E-02
GO:0010742	macrophage derived foam cell differentiation	17	29	-0.7924	3.2E-02
GO:0022407	regulation of cell-cell adhesion	17	29	-0.7924	3.2E-02
GO:0043624	cellular protein complex disassembly	43	56	-0.5231	3.3E-02
GO:0006778	porphyrin metabolic process	20	32	-0.7284	3.3E-02
GO:0000226	microtubule cytoskeleton organization	88	99	-0.3775	3.4E-02
GO:0006809	nitric oxide biosynthetic process	26	38	-0.6380	3.4E-02
GO:0007040	lysosome organization	15	26	-0.8082	3.4E-02
GO:0015671	oxygen transport	7	17	-1.1452	3.4E-02
GO:0031102	neuron projection regeneration	15	26	-0.8082	3.4E-02
GO:0032615	interleukin-12 production	15	26	-0.8082	3.4E-02
GO:0040015	negative regulation of multicellular organism growth	7	17	-1.1452	3.4E-02
GO:0045103	intermediate filament-based process	15	26	-0.8082	3.4E-02
GO:0007050	cell cycle arrest	62	74	-0.4362	3.4E-02
GO:0070265	necrotic cell death	4	13	-1.4364	3.4E-02
GO:0070723	response to cholesterol	4	13	-1.4364	3.4E-02
GO:0009225	nucleotide-sugar metabolic process	13	24	-0.8712	3.4E-02

GO:0045596	negative regulation of cell differentiation	120	128	-0.3246	3.4E-02
GO:0048598	embryonic morphogenesis	172	174	-0.2722	3.5E-02
GO:0002920	regulation of humoral immune response	9	19	-1.0052	3.5E-02
GO:0051353	positive regulation of oxidoreductase activity	18	30	-0.7691	3.5E-02
GO:0003032	detection of oxygen	3	11	-1.5569	3.5E-02
GO:0007618	mating	21	33	-0.7104	3.6E-02
GO:0009409	response to cold	144	149	-0.2944	3.6E-02
GO:0016046	detection of fungus	1	8	-2.3370	3.6E-02
GO:0043112	receptor metabolic process	34	46	-0.5609	3.6E-02
GO:0050808	synapse organization	50	62	-0.4741	3.6E-02
GO:0055094	response to lipoprotein stimulus	1	8	-2.3370	3.6E-02
GO:0032870	cellular response to hormone stimulus	80	91	-0.3883	3.6E-02
GO:0009590	detection of gravity	2	9	-1.7616	3.7E-02
GO:0034629	cellular protein complex localization	2	9	-1.7616	3.7E-02
GO:0051014	actin filament severing	2	9	-1.7616	3.7E-02
GO:0009309	amine biosynthetic process	51	63	-0.4703	3.8E-02
GO:0031503	protein complex localization	6	15	-1.1741	3.8E-02

GO:0007159	leukocyte cell-cell adhesion	19	30	-0.7150	3.9E-02
GO:0033555	multicellular organismal response to stress	25	37	-0.6505	3.9E-02
GO:0048678	response to axon injury	19	30	-0.7150	3.9E-02
GO:0031532	actin cytoskeleton reorganization	14	25	-0.8380	3.9E-02
GO:0030155	regulation of cell adhesion	78	88	-0.3800	4.0E-02
GO:0043244	regulation of protein complex disassembly	36	48	-0.5464	4.0E-02
GO:0019882	antigen processing and presentation	40	52	-0.5211	4.0E-02
GO:0019226	transmission of nerve impulse	242	234	-0.2276	4.1E-02
GO:0030865	cortical cytoskeleton organization	10	20	-0.9511	4.1E-02
GO:0045598	regulation of fat cell differentiation	10	20	-0.9511	4.1E-02
GO:0009294	DNA mediated transformation	0	5	1.79E308	4.1E-02
GO:0009956	radial pattern formation	0	5	1.79E308	4.1E-02
GO:0010157	response to chlorate	0	5	1.79E308	4.1E-02
GO:0040019	positive regulation of embryonic development	0	5	1.79E308	4.1E-02
GO:0042590	antigen processing and presentation of exogenous peptide antigen via MHC class I	0	5	1.79E308	4.1E-02
GO:0046951	ketone body biosynthetic	0	5	1.79E308	4.1E-02

	process				
GO:0051036	regulation of endosome size	0	5	1.79E308	4.1E-02
GO:0051068	dihydrolipoamide metabolic process	0	5	1.79E308	4.1E-02
GO:0060152	microtubule-based peroxisome localization	0	5	1.79E308	4.1E-02
GO:0000075	cell cycle checkpoint	45	57	-0.4953	4.1E-02
GO:0032386	regulation of intracellular transport	45	57	-0.4953	4.1E-02
GO:0046942	carboxylic acid transport	125	131	-0.3069	4.1E-02
GO:0051348	negative regulation of transferase activity	59	70	-0.4300	4.2E-02
GO:0007015	actin filament organization	110	57	-0.4699	4.2E-02
GO:0035295	tube development	119	125	-0.3091	4.2E-02
GO:0009593	detection of chemical stimulus	24	35	-0.6356	4.5E-02
GO:0042596	fear response	13	23	-0.8286	4.5E-02
GO:0006997	nucleus organization	31	42	-0.5622	4.6E-02
GO:0002645	positive regulation of tolerance induction	7	16	-1.0845	4.7E-02
GO:0008217	regulation of blood pressure	62	72	-0.4086	4.7E-02
GO:0019883	antigen processing and presentation of endogenous antigen	7	16	-1.0845	4.7E-02
GO:0042756	drinking behavior	7	16	-1.0845	4.7E-02
GO:0042554	superoxide anion	11	21	-0.9046	4.8E-02

	generation				
GO:0046689	response to mercury ion	11	21	-0.9046	4.8E-02
GO:0051354	negative regulation of oxidoreductase activity	9	18	-0.9510	4.8E-02
GO:0007292	female gamete generation	40	51	-0.5016	4.9E-02
GO:0045087	innate immune response	99	106	-0.3279	4.9E-02
GO:0042769	DNA damage response, detection of DNA damage	4	12	-1.3563	5.0E-02

[1]Over-Expressed Genes: number of genes that have a positive association between expression and glioblastoma multiforme survival.

[2]Under-Expressed Genes: number of genes that have a negative association between expression and glioblastoma multiforme survival.

[3]Log Odds Ratio: indicates whether the category is more enriched among the genes that have a positive association between expression and survival relative to the enrichment among the genes that have a negative association between expression and glioblastoma survival (positive $\log_e$ odds ratio) or vice versa (negative $\log_e$ odds ratio) . Extreme values indicate higher difference in the enrichment percentages between the positive and negative association groups meanwhile values close to zero indicate similar enrichment percentages between positive and negative association groups.

[4]FDR-adjusted P-value: False discovery rate adjusted P-value of the log odds ratio test.

Supplementary Table 5. Significant GO molecular functions (levels 3-6) from the gene set enrichment analysis of the genome.

GO Identifier	GO Molecular Function	Over-Expressed Gene[1]	Under-Expressed Genes[2]	Log Odds Ratio[3]	FDR P-value[4]
GO:0000287	magnesium ion binding	196	300	-0.6962	3.2E-11
GO:0016462	pyrophosphatase activity	417	520	-0.4962	5.2E-11
GO:0016818	hydrolase activity, acting on acid anhydrides, in phosphorus-containing anhydrid	419	521	-0.4933	5.2E-11
GO:0016817	hydrolase activity, acting on acid anhydrides	428	527	-0.4834	9.6E-11
GO:0016301	kinase activity	421	501	-0.4473	5.6E-09
GO:0016773	phosphotransferase activity, alcohol group as acceptor	393	475	-0.4624	5.6E-09
GO:0003723	RNA binding	357	437	-0.4741	9.8E-09
GO:0016788	hydrolase activity, acting on ester bonds	349	429	-0.4781	9.8E-09
GO:0016874	ligase activity	205	272	-0.5501	4.1E-07
GO:0030695	GTPase regulator activity	193	260	-0.5651	4.1E-07
GO:0016491	oxidoreductase activity	396	454	-0.4074	6.2E-07
GO:0008289	lipid binding	256	317	-0.4816	1.6E-06
GO:0042578	phosphoric ester hydrolase activity	165	224	-0.5714	2.6E-06
GO:0004672	protein kinase activity	325	380	-0.4252	2.7E-06
GO:0005543	phospholipid binding	79	132	-0.7767	3.0E-06
GO:0048037	cofactor binding	137	190	-0.5916	1.1E-05
GO:0003735	structural constituent of	273	24	-1.4861	1.3E-05

	ribosome				
GO:0008047	enzyme activator activity	169	219	-0.5242	2.6E-05
GO:0005096	GTPase activator activity	110	158	-0.6256	2.9E-05
GO:0008565	protein transporter activity	45	86	-0.9092	3.1E-05
GO:0042802	identical protein binding	317	359	-0.3919	3.2E-05
GO:0046983	protein dimerization activity	269	314	-0.4214	3.2E-05
GO:0016853	isomerase activity	71	115	-0.7446	3.9E-05
GO:0019001	guanyl nucleotide binding	204	250	-0.4687	4.1E-05
GO:0008233	peptidase activity	328	365	-0.3741	6.7E-05
GO:0019904	protein domain specific binding	160	205	-0.5122	7.1E-05
GO:0005083	small GTPase regulator activity	132	175	-0.5455	1.1E-04
GO:0016881	acid-amino acid ligase activity	104	146	-0.6021	1.1E-04
GO:0008022	protein C-terminus binding	63	100	-0.7236	2.2E-04
GO:0016791	phosphatase activity	129	169	-0.5333	2.2E-04
GO:0035091	phosphoinositide binding	49	84	-0.8001	2.5E-04
GO:0051082	unfolded protein binding	58	94	-0.7442	2.9E-04
GO:0031072	heat shock protein binding	40	73	-0.8623	3.4E-04
GO:0005536	glucose binding	1	16	-3.0308	6.0E-04
GO:0008134	transcription factor binding	251	281	-0.3778	6.0E-04
GO:0008092	cytoskeletal protein binding	270	298	-0.3638	7.3E-04

GO:0004175	endopeptidase activity	219	250	-0.3967	7.3E-04
GO:0015075	ion transmembrane transporter activity	488	489	-0.2694	1.2E-03
GO:0017124	SH3 domain binding	47	77	-0.7542	1.2E-03
GO:0019899	enzyme binding	262	287	-0.3558	1.3E-03
GO:0019787	small conjugating protein ligase activity	92	124	-0.5602	1.3E-03
GO:0050662	coenzyme binding	111	144	-0.5225	1.3E-03
GO:0016829	lyase activity	4	185	-1.7844	1.7E-03
GO:0016747	transferase activity, transferring acyl groups other than amino-acyl groups	97	127	-0.5311	2.3E-03
GO:0003756	protein disulfide isomerase activity	3	18	-2.0500	2.4E-03
GO:0008135	translation factor activity, nucleic acid binding	46	74	-0.7358	2.4E-03
GO:0008324	cation transmembrane transporter activity	392	399	-0.2835	2.4E-03
GO:0043621	protein self-association	9	28	-1.3937	2.4E-03
GO:0008415	acyltransferase activity	96	126	-0.5335	2.4E-03
GO:0003697	single-stranded DNA binding	30	55	-0.8659	2.5E-03
GO:0016746	transferase activity, transferring acyl groups	105	134	-0.5056	2.5E-03
GO:0004812	aminoacyl-tRNA ligase activity	40	66	-0.7608	2.7E-03
GO:0016876	ligase activity, forming aminoacyl-tRNA and related compounds	40	66	-0.7608	2.7E-03
GO:0003682	chromatin binding	77	106	-0.5807	2.7E-03
GO:0019208	phosphatase regulator activity	33	57	-0.8063	3.8E-03
GO:0003916	DNA topoisomerase activity	11	30	-1.2621	4.2E-03

GO:0008060	ARF GTPase activator activity	16	36	-1.0699	4.6E-03
GO:0005545	phosphatidylinositol binding	13	32	-1.1596	4.8E-03
GO:0004301	epoxide hydrolase activity	0	10	1.79E308	5.0E-03
GO:0005085	guanyl-nucleotide exchange factor activity	73	99	-0.5654	5.2E-03
GO:0008081	phosphoric diester hydrolase activity	44	69	-0.7100	5.2E-03
GO:0016830	carbon-carbon lyase activity	25	47	-0.8906	5.6E-03
GO:0030674	protein binding, bridging	46	70	-0.6798	6.1E-03
GO:0032403	protein complex binding	104	129	-0.4767	6.1E-03
GO:0046982	protein heterodimerization activity	114	138	-0.4525	7.8E-03
GO:0003918	DNA topoisomerase (ATP-hydrolyzing) activity	8	24	-1.3571	8.6E-03
GO:0043566	structure-specific DNA binding	78	102	-0.5289	8.6E-03
GO:0051536	iron-sulfur cluster binding	28	49	-0.8190	8.6E-03
GO:0016831	carboxy-lyase activity	18	37	-0.9795	9.4E-03
GO:0016868	intramolecular transferase activity, phosphotransferases	4	17	-1.7050	1.1E-02
GO:0003755	peptidyl-prolyl cis-trans isomerase activity	19	38	-0.9521	1.1E-02
GO:0051287	NAD or NADH binding	28	48	-0.7982	1.2E-02
GO:0019205	nucleobase, nucleoside, nucleotide kinase activity	26	46	-0.8298	1.2E-02
GO:0019901	protein kinase binding	77	99	-0.5118	1.2E-02

GO:0008484	sulfuric ester hydrolase activity	8	23	-1.3144	1.3E-02
GO:0042803	protein homodimerization activity	174	191	-0.3553	1.3E-02
GO:0015923	mannosidase activity	11	27	-1.1565	1.3E-02
GO:0019888	protein phosphatase regulator activity	32	52	-0.7448	1.3E-02
GO:0046332	SMAD binding	25	44	-0.8244	1.4E-02
GO:0030170	pyridoxal phosphate binding	30	50	-0.7701	1.4E-02
GO:0003743	translation initiation factor activity	33	53	-0.7331	1.4E-02
GO:0003684	damaged DNA binding	28	47	-0.7771	1.5E-02
GO:0003712	transcription cofactor activity	183	198	-0.3408	1.5E-02
GO:0004065	arylsulfatase activity	5	18	-1.5391	1.5E-02
GO:0005086	ARF guanyl-nucleotide exchange factor activity	9	24	-1.2392	1.6E-02
GO:0046906	tetrapyrrole binding	80	101	-0.4935	1.6E-02
GO:0015459	potassium channel regulator activity	14	30	-1.0207	1.7E-02
GO:0004082	bisphosphoglycerate mutase activity	0	8	1.79E30 8	1.7E-02
GO:0004619	phosphoglycerate mutase activity	0	8	1.79E30 8	1.7E-02
GO:0005516	calmodulin binding	84	104	-0.4740	1.9E-02
GO:0016810	hydrolase activity, acting on carbon-nitrogen (but not peptide) bonds	63	83	-0.5357	1.9E-02
GO:0019900	kinase binding	93	113	-0.4554	1.9E-02
GO:0008603	cAMP-dependent protein kinase regulator activity	17	33	-0.9220	2.0E-02
GO:0060090	molecular adaptor	37	56	-0.6738	2.0E-02

	activity				
GO:0004305	ethanolamine kinase activity	1	10	-2.5603	2.0E-02
GO:0005496	steroid binding	78	97	-0.4782	2.0E-02
GO:0016780	phosphotransferase activity, for other substituted phosphate groups	10	24	-1.1338	2.0E-02
GO:0016813	hydrolase activity, acting on carbon-nitrogen (but not peptide) bonds, in linear	7	20	-1.3080	2.0E-02
GO:0051635	bacterial cell surface binding	1	10	-2.5603	2.0E-02
GO:0016651	oxidoreductase activity, acting on NADH or NADPH	48	67	-0.5931	2.1E-02
GO:0004553	hydrolase activity, hydrolyzing O-glycosyl compounds	59	78	-0.5390	2.1E-02
GO:0004221	ubiquitin thiolesterase activity	36	54	-0.6647	2.2E-02
GO:0005160	transforming growth factor beta receptor binding	14	29	-0.9867	2.2E-02
GO:0017069	snRNA binding	5	17	-1.4818	2.2E-02
GO:0046527	glucosyltransferase activity	5	17	-1.4818	2.2E-02
GO:0043021	ribonucleoprotein binding	23	40	-0.8123	2.3E-02
GO:0004518	nuclease activity	88	106	-0.4464	2.4E-02
GO:0005099	Ras GTPase activator activity	47	65	-0.5837	2.4E-02
GO:0008187	poly-pyrimidine tract binding	4	15	-1.5797	2.4E-02
GO:0019887	protein kinase regulator activity	54	73	-0.5612	2.4E-02

GO:0000049	tRNA binding	13	28	-1.0257	2.4E-02
GO:0043130	ubiquitin binding	15	30	-0.9517	2.4E-02
GO:0030246	carbohydrate binding	197	206	-0.3065	2.5E-02
GO:0015267	channel activity	269	270	-0.2663	2.5E-02
GO:0003729	mRNA binding	37	54	-0.6372	2.8E-02
GO:0004417	hydroxyethylthiazole kinase activity	0	7	1.79E308	2.8E-02
GO:0004854	xanthine dehydrogenase activity	0	7	1.79E308	2.8E-02
GO:0005344	oxygen transporter activity	6	18	-1.3567	2.8E-02
GO:0008479	queuine tRNA-ribosyltransferase activity	0	7	1.79E308	2.8E-02
GO:0016655	oxidoreductase activity, acting on NADH or NADPH, quinone or similar compound as	31	48	-0.6963	2.8E-02
GO:0016814	hydrolase activity, acting on carbon-nitrogen (but not peptide) bonds, in cyclic	16	31	-0.9199	2.8E-02
GO:0017070	U6 snRNA binding	0	7	1.79E308	2.8E-02
GO:0017081	chloride channel regulator activity	0	7	1.79E308	2.8E-02
GO:0019166	trans-2-enoyl-CoA reductase (NADPH) activity	0	7	1.79E308	2.8E-02
GO:0019870	potassium channel inhibitor activity	0	7	1.79E308	2.8E-02
GO:0019992	diacylglycerol binding	37	54	-0.6372	2.8E-02
GO:0030292	protein tyrosine kinase inhibitor activity	0	7	1.79E308	2.8E-02
GO:0035254	glutamate receptor binding	7	19	-1.2566	2.8E-02

GO:0051879	Hsp90 protein binding	6	18	-1.3567	2.8E-02
GO:0003702	RNA polymerase II transcription factor activity	129	143	-0.3638	2.8E-02
GO:0003779	actin binding	182	191	-0.3097	2.9E-02
GO:0015631	tubulin binding	52	69	-0.5423	2.9E-02
GO:0016563	transcription activator activity	212	218	-0.2897	2.9E-02
GO:0016779	nucleotidyltransferase activity	73	90	-0.4693	2.9E-02
GO:0042895	antibiotic transporter activity	19	34	-0.8405	2.9E-02
GO:0008168	methyltransferase activity	9	213	-1.1139	2.9E-02
GO:0016175	superoxide-generating NADPH oxidase activity	5	16	-1.4211	2.9E-02
GO:0004014	adenosylmethionine decarboxylase activity	1	9	-2.4548	2.9E-02
GO:0016709	oxidoreductase activity, acting on paired donors, with incorporation or reductio	27	43	-0.7242	2.9E-02
GO:0042393	histone binding	27	43	-0.7242	2.9E-02
GO:0051076		1	9	-2.4548	2.9E-02
GO:0003989	acetyl-CoA carboxylase activity	2	11	-1.9625	3.0E-02
GO:0008237	metallopeptidase activity	116	130	-0.3745	3.0E-02
GO:0004835	tubulin-tyrosine ligase activity	8	20	-1.1744	3.0E-02
GO:0030247	polysaccharide binding	85	101	-0.4325	3.0E-02
GO:0008073	ornithine decarboxylase inhibitor activity	3	13	-1.7242	3.1E-02
GO:0001875	lipopolysaccharide receptor activity	4	14	-1.5106	3.2E-02

GO:0003747	translation release factor activity	4	14	-1.5106	3.2E-02
GO:0051020	GTPase binding	60	76	-0.4959	3.3E-02
GO:0008017	microtubule binding	38	54	-0.6105	3.4E-02
GO:0031406	carboxylic acid binding	89	104	-0.4158	3.5E-02
GO:0003954	NADH dehydrogenase activity	29	44	-0.6757	3.5E-02
GO:0008234	cysteine-type peptidase activity	80	95	-0.4317	3.6E-02
GO:0016776	phosphotransferase activity, phosphate group as acceptor	19	33	-0.8106	3.7E-02
GO:0019842	vitamin binding	81	96	-0.4298	3.7E-02
GO:0004659	prenyltransferase activity	9	21	-1.1054	3.7E-02
GO:0008601	protein phosphatase type 2A regulator activity	17	31	-0.8593	3.8E-02
GO:0016769	transferase activity, transferring nitrogenous groups	17	31	-0.8593	3.8E-02
GO:0016866	intramolecular transferase activity	17	31	-0.8593	3.8E-02
GO:0016798	hydrolase activity, acting on glycosyl bonds	73	88	-0.4466	3.8E-02
GO:0005044	scavenger receptor activity	28	43	-0.6878	4.0E-02
GO:0005070	SH3/SH2 adaptor activity	28	43	-0.6878	4.0E-02
GO:0031267	small GTPase binding	57	72	-0.4930	4.1E-02
GO:0050660	FAD binding	53	68	-0.5085	4.1E-02
GO:0003704	specific RNA polymerase II transcription factor activity	26	40	-0.6894	4.3E-02

GO:0004620	phospholipase activity	58	73	-0.4894	4.3E-02
GO:0005372	water transmembrane transporter activity	8	19	-1.1230	4.3E-02
GO:0050661	NADP or NADPH binding	26	40	-0.6894	4.3E-02
GO:0030165	PDZ domain binding	29	43	-0.6526	4.3E-02
GO:0003725	double-stranded RNA binding	24	38	-0.7182	4.5E-02
GO:0003857	3-hydroxyacyl-CoA dehydrogenase activity	3	12	-1.6440	4.5E-02
GO:0003913	DNA photolyase activity	0	6	1.79E308	4.5E-02
GO:0003953	NAD+ nucleosidase activity	2	10	-1.8671	4.5E-02
GO:0003975	UDP-N-acetylglucosamine-dolichyl-phosphate N-acetylglucos amine phosphotransferase	0	6	1.79E308	4.5E-02
GO:0004311	farnesyltranstransferase activity	2	10	-1.8671	4.5E-02
GO:0008320	protein transmembrane transporter activity	19	32	-0.7797	4.5E-02
GO:0008329	pattern recognition receptor activity	10	22	-1.0466	4.5E-02
GO:0008493	tetracycline transporter activity	19	32	-0.7797	4.5E-02
GO:0008521	acetyl-CoA transporter activity	0	6	1.79E308	4.5E-02
GO:0015068	glycine amidinotransferase activity	0	6	1.79E308	4.5E-02
GO:0019237	centromeric DNA binding	3	12	-1.6440	4.5E-02
GO:0030957	Tat protein binding	0	6	1.79E308	4.5E-02
GO:0035064	methylated histone	0	6	1.79E30	4.5E-02

	residue binding			8	
GO:0047016	cholest-5-ene-3-beta,7-alpha-diol 3-beta-dehydrogenase activity	2	10	-1.8671	4.5E-02
GO:0048186	inhibin beta-A binding	3	12	-1.6440	4.5E-02
GO:0004807	triose-phosphate isomerase activity	0	5	1.79E308	4.5E-02
GO:0005178	integrin binding	34	48	-0.6037	4.6E-02
GO:0005548	phospholipid transporter activity	17	30	-0.8264	4.6E-02
GO:0016594	glycine binding	7	18	-1.2025	4.6E-02
GO:0030276	clathrin binding	7	18	-1.2025	4.6E-02
GO:0030169	low-density lipoprotein binding	15	27	-0.8460	4.9E-02
GO:0043022	ribosome binding	15	27	-0.8460	4.9E-02
GO:0043498	cell surface binding	20	33	-0.7592	4.9E-02
GO:0016820	hydrolase activity, acting on acid anhydrides, catalyzing transmembrane movement	73	86	-0.4234	4.9E-02
GO:0004428	inositol or phosphatidylinositol kinase activity	23	36	-0.7065	5.0E-02
GO:0004576	oligosaccharyl transferase activity	6	16	-1.2387	5.0E-02
GO:0005504	fatty acid binding	26	39	-0.6640	5.0E-02
GO:0008034	lipoprotein binding	23	36	-0.7065	5.0E-02
GO:0009881	photoreceptor activity	11	22	-0.9512	5.0E-02
GO:0031404	chloride ion binding	43	57	-0.5409	5.0E-02
GO:0051537	2 iron, 2 sulfur cluster binding	11	22	-0.9512	5.0E-02
GO:0005319	lipid transporter activity	1	91	-2.4580	5.0E-02

[1]Over Expressed Genes: number of genes that have a positive association between expression and glioblastoma multiforme survival.

[2]Under Expressed Genes: number of genes that have a negative association between expression and glioblastoma multiforme survival.

[3]Log Odds Ratio: indicates whether the category is more enriched among the genes that have a positive association between expression and survival relative to the enrichment among the genes that have a negative association between expression and glioblastoma survival (positive $\log_e$ odds ratio) or vice versa (negative $\log_e$ odds ratio) . Extreme values indicate higher difference in the enrichment percentages between the positive and negative association groups meanwhile values close to zero indicate similar enrichment percentages between positive and negative association groups.

[4]FDR-adjusted P-value: False discovery rate adjusted P-value of the log odds ratio test.

Supplementary Table 6. Significant KEGG pathways from the gene set enrichment analysis of the genome.

KEGG Identifier	KEGG Name	Over-Expressed Gene[1]	Under-Expressed Genes[2]	Log Odds Ratio[3]	FDR P-value[4]
hsa03010	Ribosome	119	16	-2.4779	9.7E-10
hsa00010	Glycolysis / Gluconeogenesis	57	27	-1.1614	3.6E-04
hsa00190	Oxidative phosphorylation	103	39	-0.9390	3.6E-04
hsa05212	Pancreatic cancer	54	45	-0.9460	4.7E-04
hsa05130	Pathogenic Escherichia coli infection	44	41	-1.0575	4.7E-04
hsa00240	Pyrimidine metabolism	42	78	-0.8800	5.0E-04
hsa03050	Proteasome	33	32	-1.0965	7.2E-04
hsa00280	Valine, leucine and isoleucine degradation	20	48	-1.1353	8.5E-04
hsa04662	B cell receptor signaling pathway	34	65	-0.9084	8.5E-04
hsa05223	Non-small cell lung cancer	25	52	-0.9922	9.0E-04
hsa05120	Epithelial cell signaling in Helicobacter pylori infection	30	59	-0.9365	9.0E-04
hsa00020	Citrate cycle (TCA cycle)	15	38	-1.1888	1.1E-03
hsa04012	ErbB signaling pathway	44	72	-0.7528	1.3E-03
hsa04070	Phosphatidylinositol signaling system	37	64	-0.8080	1.3E-03

hsa04664	Fc epsilon RI signaling pathway	38	65	-0.7969	1.3E-03
hsa04370	VEGF signaling pathway	35	61	-0.8155	1.6E-03
hsa05222	Small cell lung cancer	44	70	-0.7244	1.8E-03
hsa05220	Chronic myeloid leukemia	38	63	-0.7655	2.0E-03
hsa00970	Aminoacyl-tRNA biosynthesis	21	43	-0.9760	2.1E-03
hsa05211	Renal cell carcinoma	35	59	-0.7820	2.3E-03
hsa05012	Parkinson's disease	59	85	-0.6256	2.4E-03
hsa00760	Nicotinate and nicotinamide metabolism	12	31	-1.2079	2.4E-03
hsa00565	Ether lipid metabolism	17	37	-1.0367	2.4E-03
hsa05014	Amyotrophic lateral sclerosis (ALS)	28	50	-0.8393	2.7E-03
hsa05410	Hypertrophic cardiomyopathy	43	66	-0.6883	3.0E-03
hsa00071	Fatty acid metabolism	21	41	-0.9282	3.0E-03
hsa00310	Lysine degradation	23	43	-0.8849	3.0E-03
hsa00030	Pentose phosphate pathway	15	34	-1.0772	3.0E-03
hsa04540	Gap junction	47	70	-0.6583	3.2E-03
hsa03040	Spliceosome	67	90	-0.5555	3.8E-03
hsa00562	Inositol phosphate metabolism	30	51	-0.7900	3.8E-03

hsa00650	Butanoate metabolism	15	33	-1.0473	3.8E-03
hsa04670	Leukocyte transendothelial migration	61	83	-0.5681	4.0E-03
hsa05412	Arrhythmogenic right ventricular cardiomyopathy	40	61	-0.6816	4.3E-03
hsa00340	Histidine metabolism	16	34	-1.0126	4.3E-03
hsa04940	Type I diabetes mellitus	20	38	-0.9008	4.3E-03
hsa04210	Apoptosis	47	68	-0.6291	4.5E-03
hsa04640	Hematopoietic cell lineage	45	66	-0.6427	4.8E-03
hsa05213	Endometrial cancer	29	48	-0.7631	5.3E-03
hsa05210	Colorectal cancer	66	44	-0.7220	5.7E-03
hsa05218	Melanoma	38	57	-0.6648	7.4E-03
hsa05414	Dilated cardiomyopathy	48	67	-0.5931	7.4E-03
hsa05221	Acute myeloid leukemia	32	50	-0.7054	7.4E-03
hsa00510	N-Glycan biosynthesis	27	45	-0.7699	7.4E-03
hsa00564	Glycerophospholipid metabolism	36	54	-0.6647	7.8E-03
hsa04920	Adipocytokine signaling pathway	37	55	-0.6557	8.2E-03
hsa00770	Pantothenate and CoA biosynthesis	11	25	-1.0793	8.2E-03
hsa00380	Tryptophan metabolism	21	37	-0.8251	8.2E-03
hsa00511	Other glycan degradation	8	21	-1.2233	8.3E-03

hsa04520	Adherens junction	42	60	-0.6160	9.2E-03
hsa04720	Long-term potentiation	39	57	-0.6388	9.3E-03
hsa05219	Bladder cancer	25	41	-0.7535	9.4E-03
hsa00670	One carbon pool by folate	7	19	-1.2566	9.4E-03
hsa05214	Glioma	38	55	-0.6289	1.0E-02
hsa00531	Glycosaminoglycan degradation	11	24	-1.0384	1.1E-02
hsa03320	PPAR signaling pathway	40	56	-0.5956	1.2E-02
hsa05310	Asthma	18	32	-0.8339	1.3E-02
hsa04150	mTOR signaling pathway	29	44	-0.6757	1.3E-02
hsa04621	NOD-like receptor signaling pathway	35	51	-0.6355	1.3E-02
hsa03018	RNA degradation	32	48	-0.6644	1.3E-02
hsa04666	Fc gamma R-mediated phagocytosis	50	66	-0.5370	1.4E-02
hsa05215	Prostate cancer	51	67	-0.5322	1.4E-02
hsa00330	Arginine and proline metabolism	31	46	-0.6535	1.5E-02
hsa05110	Vibrio cholerae infection	31	46	-0.6535	1.5E-02
hsa00410	beta-Alanine metabolism	13	26	-0.9515	1.5E-02
hsa00250	Alanine, aspartate and glutamate metabolism	20	34	-0.7892	1.5E-02

hsa03020	RNA polymerase	16	29	-0.8531	1.6E-02
hsa00561	Glycerolipid metabolism	27	41	-0.6764	1.7E-02
hsa04130	SNARE interactions in vesicular transport	24	38	-0.7182	1.7E-02
hsa00072	Synthesis and degradation of ketone bodies	3	12	-1.6440	1.7E-02
hsa04912	GnRH signaling pathway	57	71	-0.4789	1.8E-02
hsa04710	Circadian rhythm	9	20	-1.0565	1.9E-02
hsa04350	TGF-beta signaling pathway	48	62	-0.5151	2.1E-02
hsa05330	Allograft rejection	20	32	-0.7284	2.6E-02
hsa05340	Primary immunodeficiency	23	35	-0.6783	2.7E-02
hsa00062	Fatty acid elongation in mitochondria	4	13	-1.4364	2.7E-02
hsa00785	Lipoic acid metabolism	3	11	-1.5569	2.8E-02
hsa04914	Progesterone-mediated oocyte maturation	51	63	-0.4703	3.0E-02
hsa00640	Propanoate metabolism	19	30	-0.7150	3.0E-02
hsa00620	Pyruvate metabolism	25	37	-0.6505	3.0E-02
hsa04115	p53 signaling pathway	39	51	-0.5270	3.0E-02
hsa00350	Tyrosine metabolism	28	40	-0.6152	3.0E-02
hsa00260	Glycine, serine and threonine metabolism	19	30	-0.7150	3.0E-02
hsa00533	Keratan sulfate	12	22	-0.8641	3.1E-02

	biosynthesis				
hsa00120	Primary bile acid biosynthesis	12	22	-0.8641	3.1E-02
hsa04614	Renin-angiotensin system	10	20	-0.9511	3.1E-02
hsa04612	Antigen processing and presentation	85	26	-0.7224	3.1E-02
hsa00860	Porphyrin and chlorophyll metabolism	24	35	-0.6356	3.5E-02
hsa00630	Glyoxylate and dicarboxylate metabolism	9	18	-0.9510	3.8E-02
hsa04620	Toll-like receptor signaling pathway	59	69	-0.4156	3.9E-02
hsa00512	O-Glycan biosynthesis	20	30	-0.6636	4.2E-02
hsa03440	Homologous recombination	20	30	-0.6636	4.2E-02
hsa03430	Mismatch repair	17	27	-0.7207	4.2E-02
hsa00910	Nitrogen metabolism	15	25	-0.7689	4.5E-02

[1]Over-Expressed Genes: number of genes that have a positive association between expression and glioblastoma multiforme survival.

[2]Under-Expressed Genes: number of genes that have a negative association between expression and glioblastoma multiforme survival.

[3]Log Odds Ratio: indicates whether the category is more enriched among the genes that have a positive association between expression and survival relative to the enrichment among the genes that have a negative association between expression and glioblastoma survival (positive log_e odds ratio) or vice versa (negative log_e odds ratio) . Extreme values indicate higher difference in the enrichment percentages between the positive and negative association groups meanwhile values close to zero indicate similar enrichment percentages between positive and negative association groups.

[4]FDR-adjusted P-value: False discovery rate adjusted P-value of the log odds ratio test.
