# Supplementary material for: Identification and characterization of alternative exon usage linked glioblastoma multiforme survival
Source: BMC Med Genomics. 2012 Dec 4;5:59. doi: 10.1186/1755-8794-5-59 (PMC3548711; doi:10.1186/1755-8794-5-59)
Supplement: Additional file 2 — Figure S1. Anapc1 exon expression, moving average, and alternative splicing models. Depicts the alternative exon expression, moving average and alternative splicing models for anaphase promoting complex subunit 1. Figure S2. Herc2 exon expression, moving average, and alternative splicing models. Depicts the alternative exon expression, moving average and alternative splicing models for HECT domain and RLD domain containing E3 ubiquitin protein ligase 2. [file 1755-8794-5-59-S2.pdf]

Supplementary Figure 1. *Anapc1* exon expression, moving average, and alternative splicing models.

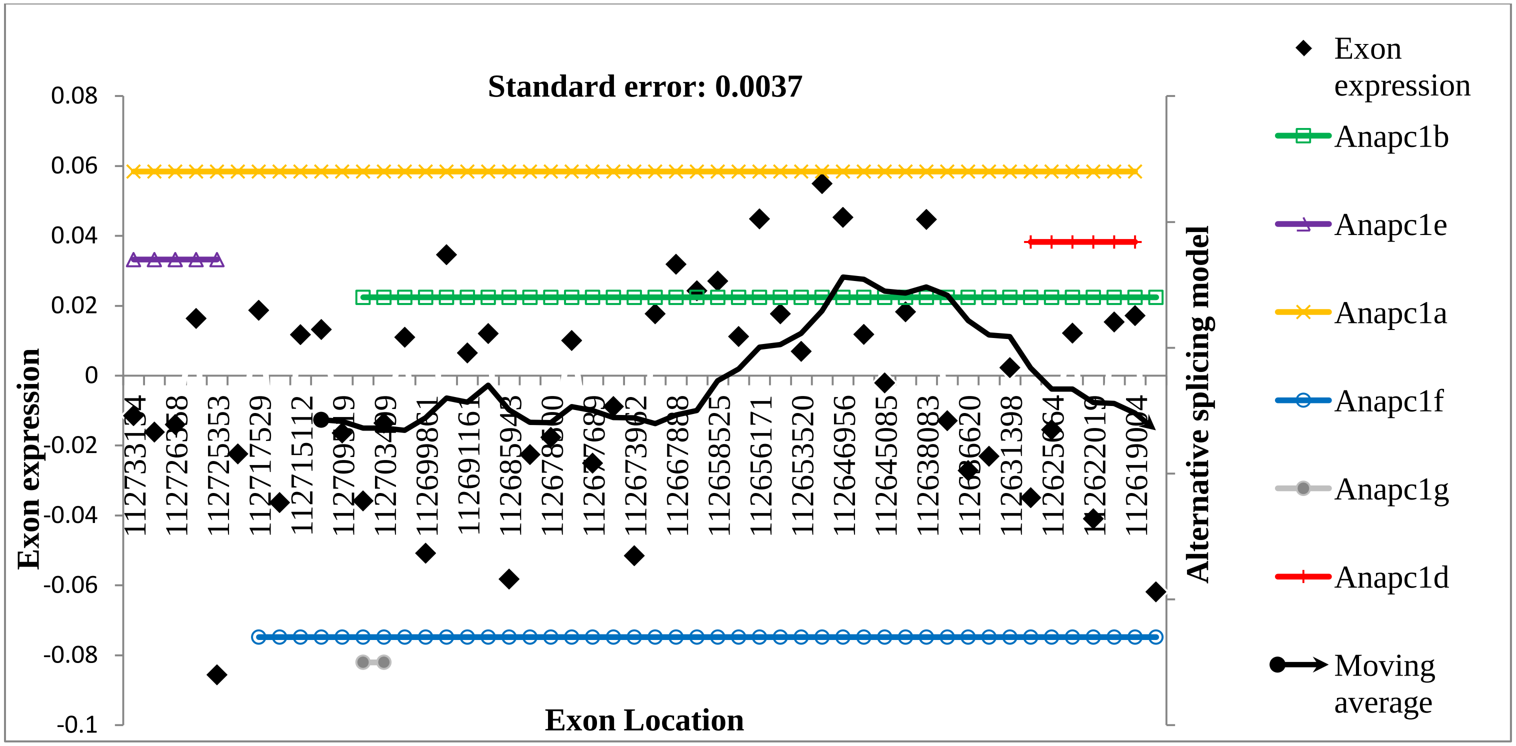

***Anapc1*: anaphase promoting complex subunit 1.**
X-axis: location in the gene (in bp).
Y-axis (left): change in exon expression per additional survival month calculated from the alternative exon usage model. Full diamond black markers: exon expression from the alternative exon usage model (Exon expression). Continuous black line: moving average pattern of expression based on 10 exons. Standard Error: standard error of the exon expression estimate.
Right Y-axis: indicator of AceView alternative splicing model. Colored continuous and dotted lines including cross, triangle, square, circle, line, and plus markers: indicator of the location of the AceView alternative splicing models (AceView models indicate exon series or cassette locations in the gene).

Anapc1 is located on chromosome 2 and the function of this gene is associated with transition in the cell cycle from metaphase to anaphase [1]. In agreement with the function, premature truncation of the gene leading to reduced expression of *Anapc1* is associated with cancer development [2]. Six AS models for this gene were found in the alternative transcript variant database AceView. *Anapc1*

exhibited AEU in this study and, of the 48 exons analyzed, the expression of 25 exons were associated with GBM survival (Supplementary Figure 1). The AS pattern predicted by our model and highlighted by the moving average trend is supported by AS gene models (Anapc1.dDec03, and Anapc1.edec03, Supplementary Figure 1). Our model predicted under-expression of the majority of the exons in three AceView AS models (Anapc1.dDec03, Anapc1.edec03 and Anapc1.gDec03) compared to the other exons. The under-expression of these exons associated with higher survival predicted by our model and presented in Supplementary Figure 1 are in consistent with previous studies of the relationship between *Anapc1* and cancer [2]. Consistent with the functional analysis, Anapc1 pertains to enriched GO biological process of cell cycle phase and axonogenesis and the KEGG pathway Ubiquitin mediated proteolysis. For *Anapc1*, the relative difference in R^2 between the training and validation data sets was 12.5%. The Pearson correlation of the exon-survival associations between the training and validation data sets was 91.3%.

Supplementary Figure 2. *Herc2* exon expression, moving average, and alternative splicing models.

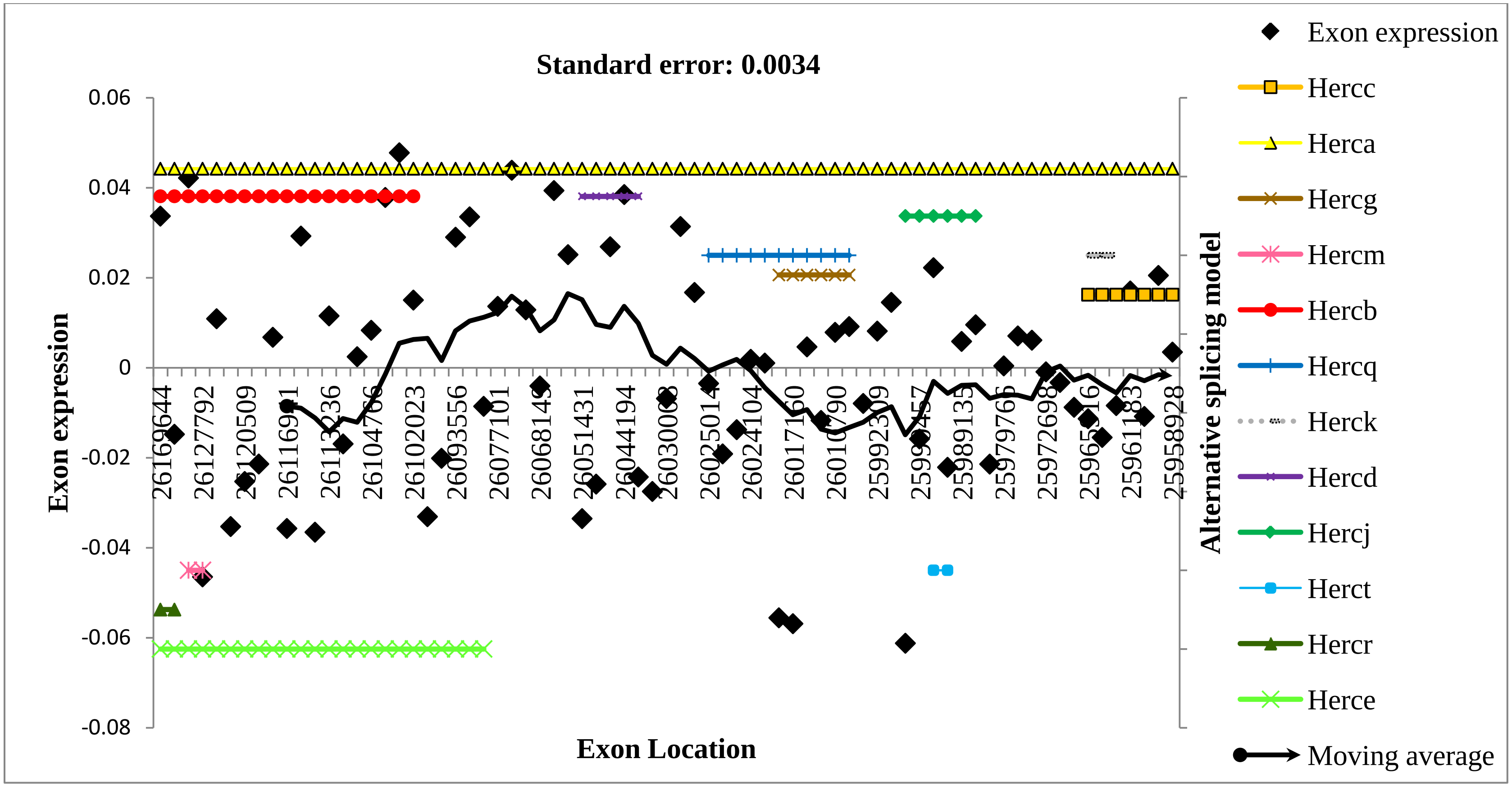

Herc2: **HECT domain and RLD domain containing E3 ubiquitin protein ligase 2.**

X-axis: location in the gene (in bp).

Y-axis (left): change in exon expression per additional survival month calculated from the alternative exon usage model. Full diamond black markers: exon expression from the alternative exon usage model (Exon expression). Continuous black line: moving average pattern of expression based on 10 exons. Standard Error: standard error of the exon expression estimate.

Right Y-axis: indicator of AceView alternative splicing model. Colored continuous and dotted lines including cross, triangle, square, circle, line, and plus markers: indicator of the location of the AceView alternative splicing models (AceView models indicate exon series or cassette locations in the gene).

Herc2 is located on chromosome 15 and belongs to the ubiquitin ligase family HERC. Various members of this family have high expression in fetal relative to adult brain [3]. *Herc2* in mouse has been associated with neuromuscular disorder, and has been proposed to be related to neuronal tissues in humans. Also, mutations resulting in under expression of Herc2 have been related to gastric and colorectal carcinomas [4]. Significant AEU and association between GBM survival and expression were detected on 42 of the 93 exons in *Herc2* (Supplementary Figure 2). Our model predicted exon under-expression that overlap with several AceView AS models (e.g. qdec03, gdec03, jdec03 and tdec03). These trends are consistent with demonstrations that HERC2 depletion restores the breast cancer suppressor BRCA1 [5], and that resulting HERC2 protein formation and cancer [4]. Supporting our functional analyses results and enriched categories, *Herc2* belongs to the GO molecular function categories GTPase regulator activity and ion binding, the GO biological process of intracellular transport and protein localization, and the KEGG pathway Ubiquitin mediated proteolysis. For *Herc2*, the relative difference in R^2 between the training and validation data sets was 10.1%. The Pearson correlation of the exon-survival associations between the training and validation data sets was 92.9%.

References

1. Jorgensen PM, Graslund S, Betz R, Stahl S, Larsson C, Hoog C: **Characterisation of the human APC1, the largest subunit of the anaphase-promoting complex.** Gene 2001, **262**(1-2):51-59.

2. He ML, Chen Y, Chen Q, He Y, Zhao J, Wang J, Yang H, Kung HF: **Multiple gene dysfunctions lead to high cancer-susceptibility: evidences from a whole-exome sequencing study.** Am J Cancer Res 2011, **1**(4):562-573.

3. Hochrainer K, Mayer H, Baranyi U, Binder B, Lipp J, Kroismayr R: **The human HERC family of ubiquitin ligases: novel members, genomic organization, expression profiling, and evolutionary aspects.** Genomics 2005, **85**(2):153-164.

4. Xu L, Drachenberg C, Burke A: **Intimal IgM lambda paraprotein deposition in myocardial arteries resulting in acute myocardial infarction and sudden death.** Pathology 2011, **43**(7):732-734.

5. Wu W, Sato K, Koike A, Nishikawa H, Koizumi H, Venkitaraman AR, Ohta T: **HERC2 is an E3 ligase that targets BRCA1 for degradation.** Cancer Res 2010, **70**(15):6384-6392.
